# Silencing miR-370-3p rescues funny current and sinus node function in heart failure

**DOI:** 10.1038/s41598-020-67790-0

**Published:** 2020-07-09

**Authors:** Joseph Yanni, Alicia D’Souza, Yanwen Wang, Ning Li, Brian J. Hansen, Stanislav O. Zakharkin, Matthew Smith, Christina Hayward, Bryan A. Whitson, Peter J. Mohler, Paul M. L. Janssen, Leo Zeef, Moinuddin Choudhury, Min Zi, Xue Cai, Sunil Jit R. J. Logantha, Shu Nakao, Andrew Atkinson, Maria Petkova, Ursula Doris, Jonathan Ariyaratnam, Elizabeth J. Cartwright, Sam Griffiths-Jones, George Hart, Vadim V. Fedorov, Delvac Oceandy, Halina Dobrzynski, Mark R. Boyett

**Affiliations:** 1 Division of Cardiovascular Sciences, University of Manchester, 46 Grafton Street, Manchester, M13 9NT UK; 2 Physiology and Cell Biology, Ohio State University Wexner Medical Center, Columbus, OH 43210 USA; 3 Bob and Corrine Frick Center for Heart Failure and Arrhythmia Research and Dorothy M. Davis Heart and Lung Research Institute, Ohio State University, Columbus, OH 43210 USA; 4 Department of Surgery, Division of Cardiac Surgery, Ohio State University Wexner Medical Center, Columbus, OH 43210 USA; 5 Department of Internal Medicine, Ohio State University Wexner Medical Center, Columbus, OH 43210 USA; 6 Bioinformatics Core Facility, University of Manchester, Manchester, UK; 7 Liverpool Centre for Cardiovascular Science, University of Liverpool, Liverpool, UK; 8 Division of Evolution and Genomics Sciences, University of Manchester, Manchester, UK; 9 Department of Anatomy, Jagiellonian University Medical College, Kraków, Poland; 10 Department of Biomedical Sciences, Faculty of Health and Medical Sciences, University of Copenhagen, 2200N Copenhagen, Denmark

**Keywords:** Heart failure, Cardiac hypertrophy

## Abstract

Bradyarrhythmias are an important cause of mortality in heart failure and previous studies indicate a mechanistic role for electrical remodelling of the key pacemaking ion channel HCN4 in this process. Here we show that, in a mouse model of heart failure in which there is sinus bradycardia, there is upregulation of a microRNA (miR-370-3p), downregulation of the pacemaker ion channel, HCN4, and downregulation of the corresponding ionic current, *I*_f_, in the sinus node. In vitro, exogenous miR-370-3p inhibits HCN4 mRNA and causes downregulation of HCN4 protein, downregulation of *I*_f_, and bradycardia in the isolated sinus node. In vivo, intraperitoneal injection of an antimiR to miR-370-3p into heart failure mice silences miR-370-3p and restores HCN4 mRNA and protein and *I*_f_ in the sinus node and blunts the sinus bradycardia. In addition, it partially restores ventricular function and reduces mortality. This represents a novel approach to heart failure treatment.

## Introduction

Heart failure is a major health problem affecting ~ 26 million people worldwide and is one of the leading causes of hospitalisation in USA and Europe, resulting in over 1 million admissions/year as a primary diagnosis^1^. Heart failure patients often have a relatively fast heart rate (> 70 beats/min)^2,3^. β-blocker and ivabradine treatment received by heart failure patients lowers the heart rate^4,5^, which may or may not be the mechanism underlying the protective effects of these medications^4^. Nevertheless, and paradoxically, bradyarrhythmias (excessively slow heart rates and rhythms) account for up to half of the deaths of end-stage heart failure patients^6–8^. In the human and animal models, there is dysfunction of the pacemaker of the heart, the sinus node, in heart failure: there is a decrease in the intrinsic heart rate (the heart rate set by the sinus node in the absence of autonomic nerve activity) and an increase in the corrected sinus node recovery time (a frequently used measure of the functioning of the sinus node)^9–13^. The dysfunction of the sinus node in heart failure has been attributed to fibrosis^14^ and also remodelling of ion channels and corresponding ionic currents underlying the functioning of the sinus node^10,11,15,16^. In particular, it has been attributed to a downregulation of the important pacemaker ion channel, HCN4, and the corresponding funny current, *I*_f_^11,15^; however, the cause of the remodelling is not known.

MicroRNAs (or miRs) are a class of abundant, non-coding RNA of 19–25 nucleotides length and are evolutionarily conserved regulatory molecules^17^. MicroRNAs negatively regulate gene expression at the post-transcriptional level by translational repression or degradation of mRNA^18^. 2,588 mature microRNAs are listed in ‘miRBase’ for *Homo sapiens*^19^. MicroRNAs are central players in gene regulation: computational prediction suggests that > 60% of all mammalian protein-coding genes are conserved targets of microRNAs, while every microRNA can potentially target hundreds of different genes^20^. MicroRNAs have been implicated in cardiac development, cardiac arrhythmias, myocardial infarction and pressure overload-induced cardiac hypertrophy^21–23^. For example, patients with unstable angina pectoris show a highly significant upregulation of various microRNAs including miR-370 in the blood compared to those with stable angina pectoris^24^. Elevated expression of miR-370 can possibly be used to stratify coronary artery disease patients that are at risk of acute coronary events^25^. In another study, miR-370 levels in the blood were found to be significantly higher in patients with type 2 diabetes and coronary artery disease^26^.

Here we show a new role for miR-370-3p. We show that in a mouse model of heart failure there is an upregulation of miR-370-3p in the sinus node (but not in ventricular muscle) and this results in a downregulation of HCN4 and *I*_f_ and consequently sinus bradycardia. In vivo an antimiR (a small molecule that silences a particular microRNA) to miR-370-3p blunts the heart failure-induced sinus bradycardia. In addition, it also partially restores whole heart function (e.g. left ventricle fractional shortening), restores body weight and reduces mortality.

## Results

### Heart failure produces sinus bradycardia and first-degree heart block

**Fig.1:**
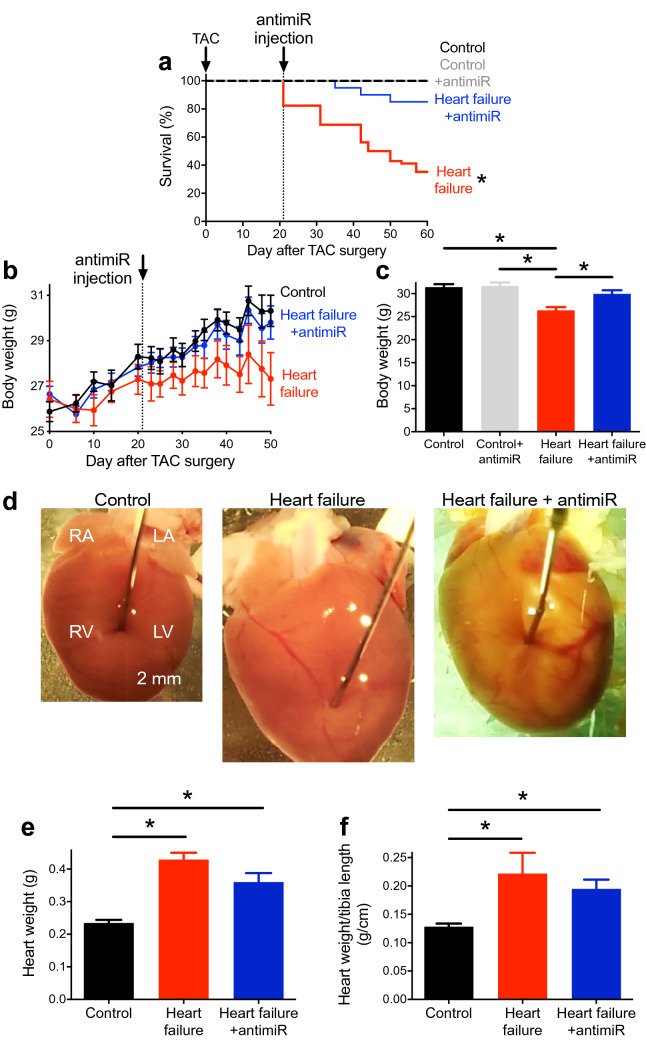
Silencing miR-370-3p improves mortality and heart weight in heart failure. (**a**) Percentage survival of control mice given PBS (black trace; subject to sham operation; n = 14), heart failure mice given PBS (red trace, subject to TAC surgery; n = 17), control mice given antimiR-370-3p (grey trace; subject to sham operation; n = 5) and heart failure mice given antimiR-370-3p (blue trace, subject to TAC surgery; n = 20) following TAC surgery. *P* < 0.05; log-rank (Mantel-Cox) test. (**b**) Body weight of control mice given PBS, heart failure mice given PBS and heart failure mice given antimiR-370-3p following TAC surgery (n = 9, 5 and 10). (**c**) Body weight on day of sacrifice of control mice given PBS, control mice given antimiR-370-3p, heart failure mice given PBS and heart failure mice given antimiR-370-3p (n = 15, 6, 15 and 10). *P < 0.05; one-way ANOVA followed by Tukey’s multiple comparisons test. (**d**) Typical examples of hearts from a control mouse given PBS, heart failure mouse given PBS, and heart failure mouse given antimiR-370-3p. All images shown on the same scale. LA, left atrium; LV, left ventricle, RA, right atrium; RV, right ventricle. (**e**, **f**) Heart weight (n = 16, 19 and 10) and heart weight to tibia length ratio (n = 8, 4 and 10) of the three groups of mice. *P < 0.05; one-way ANOVA followed by Tukey’s multiple comparisons test.

**Figure 2:**
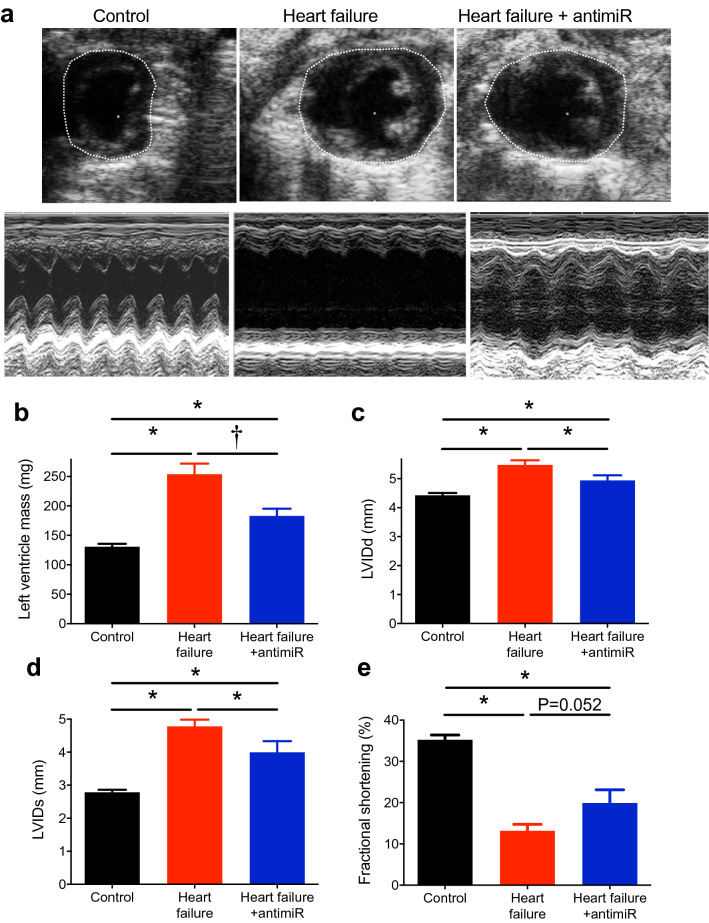
Silencing miR-370-3p improves cardiac function in heart failure. (**a**) Cross section images during diastole (all shown on the same scale) and M-mode images (all shown on the same scale) of the left ventricle in a control mouse given PBS, heart failure mouse given PBS and heart failure mouse given antimiR-370-3p. (**b**) Mean left ventricle mass (n = 19, 12 and 10). *P < 0.05; Kruskal–Wallis test followed by Dunn’s multiple comparisons test. ^†^*P* < 0.05; Mann–Whitney test. (**c**–**e**) Left ventricle inner dimension during diastole (LVIDd; n = 19, 13 and 10), inner dimension during systole (LVIDs; n = 19, 13 and 10) and fractional shortening (n = 19, 13 and 8) in the three groups of mice. *P < 0.05; one-way ANOVA followed by Tukey’s multiple comparisons test.

In mice, transverse aortic constriction (TAC) was used to produce pressure overload-induced heart failure (a commonly used experimental model^27^). From 20 days following TAC surgery, mice began showing characteristic signs of heart failure and on reaching pre-defined end-points (e.g. loss of more than 20% of body weight) had to be sacrificed (Fig. 1a). 60 days after surgery, 65% of the heart failure mice had reached the end points, whereas none of the control (sham-operated) mice had (Fig. 1a). Compared with the control mice, heart failure mice had a reduced body weight (Fig. 1b, c), larger, heavier hearts (hypertrophied; Fig. 1d, e) and a greater heart weight to tibia length ratio as well as heart weight to body weight ratio (Fig. 1f; Supplementary Fig. 1a). Echocardiography showed an enlargement of the left ventricle in the heart failure mice as compared with the control mice (Fig. 2a); this was confirmed by significant increases in left ventricle mass and internal dimension during diastole (LVIDd) and systole (LVIDs) in the heart failure mice (Fig. 2b–d; Supplementary Table 1). In the heart failure mice, left ventricular contractile dysfunction was prominent—there was reduced left ventricle fractional shortening and ejection fraction (Fig. 2a, e; Supplementary Fig. 1b, c; Supplementary Table 1). The left ventricle fractional shortening was low in this study (Fig. 2e; in the conscious mouse it is ~ 60%), but consistent with that in other studies on the anaesthetised mouse^28^.

**Figure 3:**
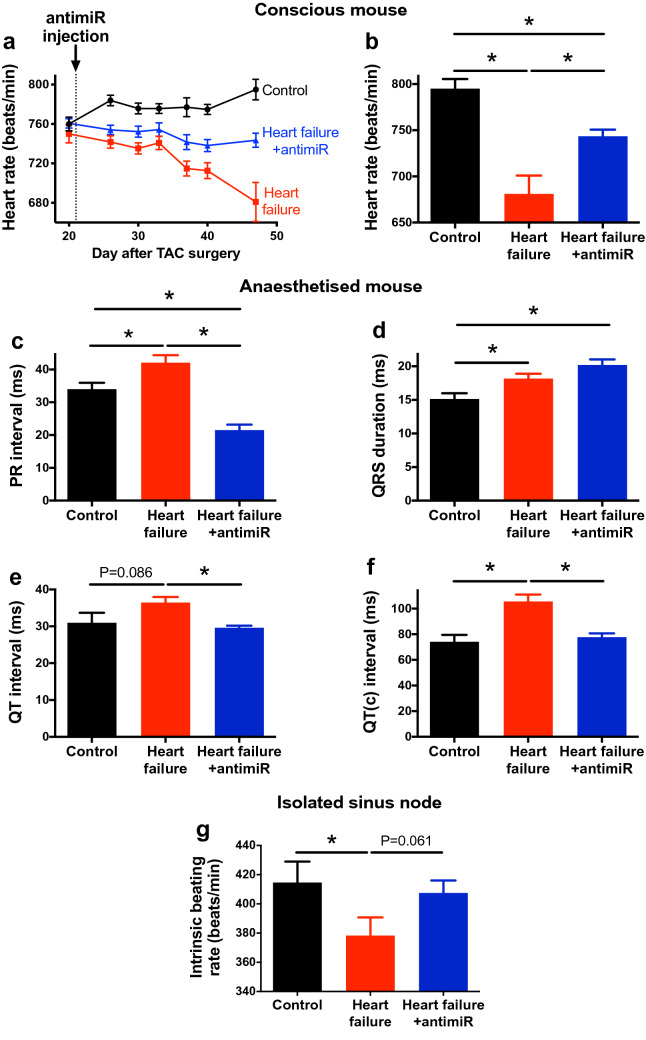
Silencing miR-370-3p improves ECG parameters in heart failure. (**a**) Heart rate (measured in conscious animals) of control mice given PBS, heart failure mice given PBS and heart failure mice given antimiR-370-3p following TAC surgery (n = 13, 13 and 17). (**b**) Heart rate (measured in conscious animals) of the three groups of mice on the day of sacrifice (n = 8, 9 and 16). *P < 0.05; one-way ANOVA followed by Tukey’s multiple comparisons test. (**c**–**f**) PR interval (n = 13, 10 and 8), QRS duration (n = 9, 14 and 9), QT interval (n = 9, 14 and 9) and corrected QT interval (n = 9, 14 and 9) of the three groups of mice on the day of sacrifice (measured in anaesthetised animals). *P < 0.05; one-way ANOVA followed by Tukey’s multiple comparisons test. (**g**) Intrinsic heart rate (measured from the isolated sinus node preparation) of the three groups of mice (n = 8, 11 and 13). *P < 0.05; one-way ANOVA followed by Holm-Sidak’s test (one comparison only). The difference between heart failure and heart failure + antimir was tested using Student’s unpaired t test (P value given).

The heart rate was recorded in vivo non-invasively from unrestrained and conscious mice using an ECGenie; there was a sinus bradycardia in the heart failure mice (the heart rate was significantly slower than that of the control mice) and the bradycardia worsened with time (Fig. 3a, b). The ECG was recorded in vivo in the anaesthetised mouse. In the heart failure mice, there was a significant increase of the PR interval (first-degree heart block) and QRS duration, indicating slowing of conduction through the atrioventricular node and His-Purkinje system (Fig. 3c, d), two other characteristics of heart failure^29^. There was also an increase in the QT and corrected QT intervals (Fig. 3e, f), again characteristic of heart failure^30^. In vivo, the heart rate is influenced by the autonomic nervous system and it is also important to measure the intrinsic heart rate (in the absence of any such influence). This was measured in isolated sinus node preparations and the intrinsic heart rate was significantly slower in the heart failure mice (Fig. 3g). Together, these data demonstrate that in the mouse TAC model of heart failure there is dysfunction of all three parts of the cardiac conduction system (sinus node, atrioventricular node and His-Purkinje system) as there is in heart failure patients^29^. In heart failure, contrary to the bradycardia observed in the conscious mouse and in the isolated sinus node preparation (Fig. 3a, g), a relative tachycardia was observed in vivo in the anaesthetised mouse (Supplementary Fig. 2). The apparent tachycardia is consistent with previous studies using the TAC model and may be the result of the well-known effect of anaesthetic on the autonomic nervous system and cardiac ion channels^31,32^.

### Heart failure produces remodelling of the sinus node

**Figure 4:**
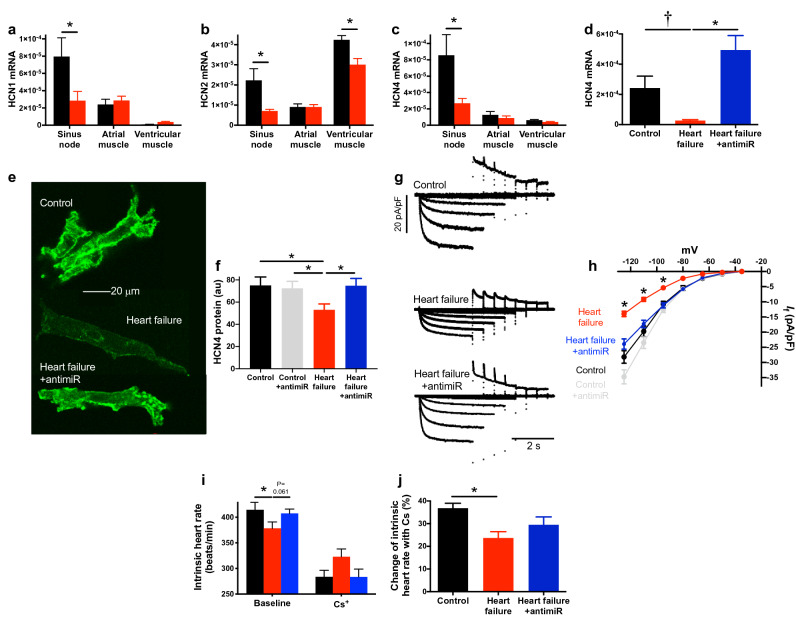
Silencing miR-370-3p improves functioning of the sinus node in heart failure. (**a**–**c**) Expression of mRNA for HCN1 (a; n = 7, 8, 6, 10, 6 and 10), HCN2 (b; n = 7, 8, 6, 10, 6 and 10) and HCN4 (c; n = 7, 8, 5, 9, 6 and 9) in the sinus node, atrial muscle and ventricular muscle of control and heart failure mice. *P < 0.05; one-way ANOVA followed by Sidak’s multiple comparisons test. (**d**) Expression of HCN4 mRNA in the sinus node of control mice given PBS, heart failure mice given PBS and heart failure mice given antimiR-370-3p (n = 14, 8 and 10). *P < 0.05; Kruskal–Wallis test followed by Dunn’s multiple comparisons test. ^†^*P* < 0.05; Mann–Whitney test. (**e**) Sinus node cells from the three types of mouse immunolabelled for HCN4. All images at the same scale. (**f**) Mean intensity of immunolabelling of HCN4 in sinus node cells from control mice given PBS, control mice given antimiR-370-3p, heart failure mice given PBS and heart failure mice given antimiR-370-3p (n = 26, 40, 39 and 22 cells). *P < 0.05; Kruskal–Wallis test followed by Dunn’s multiple comparisons test. (**g**) Families of recordings of *I*_f_ from sinus node cells from control mice given PBS, heart failure mice given PBS and heart failure mice given antimiR-370-3p. (**h**) Mean current–voltage relationships for *I*_f_ from sinus node cells from control mice given PBS, control mice given antimiR-370-3p, heart failure mice given PBS and heart failure mice given antimiR-370-3p (n = 11, 43, 40 and 46 cells). *P < 0.05; significant differences between heart failure mice given PBS and (1) control mice given PBS, (2) heart failure mice given antimiR-370-3p and (3) control mice given antimiR-370-3p; other significant differences are not shown for clarity. (**i**) Intrinsic heart rate (measured from the isolated sinus node preparation) of control mice given PBS, heart failure mice given PBS and heart failure mice given antimiR-370-3p under baseline conditions (same data as in Fig. 3g) and in the presence of 2 mM Cs^+^ to block *I*_f_; see Fig. 3g legend for n numbers and meaning of symbols. (**j**) Percentage change in the intrinsic heart rate of control mice given PBS, heart failure mice given PBS and heart failure mice given antimiR-370-3p on the addition of 2 mM Cs^+^ (n = 16, 15 and 14). *P < 0.05; one-way ANOVA followed by Tukey’s multiple comparisons test.

The sinus bradycardia could be the result of a remodelling of ion channels and the corresponding ionic currents in the sinus node^10,11,15^, or the result of fibrosis in the sinus node^14^. Tissue biopsies were taken of sinus node as well as atrial and ventricular muscle and the expression of a selection of transcripts was measured by quantitative PCR (qPCR). In the sinus node of the heart failure mice, of the transcripts targeted, some were significantly upregulated, some unchanged and some significantly downregulated (Supplementary Figs. 3 and 4). There are two principal pacemaker mechanisms in the sinus node, the membrane clock and the intracellular Ca^2+^ clock^33^. In the sinus node of the heart failure mice, there was no change in the mRNA for various components of the intracellular Ca^2+^ clock (RyR2, SERCA2, calsequestrin and NCX1; Supplementary Figs. 3 and 5) and no change in the mRNA for related Ca^2+^ channel subunits (Ca_v_1.2, Ca_v_α2δ2; Supplementary Fig. 3). These data suggest that there is no change in the sinus node intracellular Ca^2+^ clock in heart failure (consistent with the findings of Verkerk et al.^34^). However, this conclusion is tentative, because it can be argued that the data may be affected by atrial muscle contaminating the sinus node biopsies (although previously we have shown that atrial cell markers are poorly expressed in sinus node biopsies, whereas the reverse is true for sinus node markers^35,36^). The intracellular Ca^2+^ clock is not considered further. The most important component of the membrane clock is the funny current (*I*_f_)^37^, for which HCN1, HCN2 and in particular HCN4 are responsible^38^. There was a significant downregulation of mRNA for each of the HCN isoforms in the sinus node of the heart failure mice (Fig. 4a–c). Sinus node cells were isolated from control and heart failure mice and immunolabelled for HCN4 protein (Fig. 4e) and image analysis showed a statistically significant decrease in HCN4 protein immunolabelling in the heart failure mice (Fig. 4f). There should be a corresponding downregulation of *I*_f_ and this was tested using the patch clamp technique and isolated sinus node cells. There was a small but significant downregulation in the amplitude of *I*_f_ in heart failure (Supplementary Fig. 6). However, in the Supplementary Discussion we argue that the density rather than amplitude of *I*_f_ is important. In heart failure, sinus node cells were larger and had a higher capacitance and consequently the downregulation in the density of *I*_f_ (Fig. 4g) was larger than the downregulation in amplitude in heart failure (Supplementary Fig. 6). This is confirmed by data presented in Fig. 4h, which shows mean current–voltage relationships for *I*_f_ density; the decrease was 50% at − 125 mV. To test whether this decrease in *I*_f_ is responsible for the decrease in the intrinsic heart rate in heart failure, the intrinsic heart rate (measured in the isolated sinus node) was measured under baseline conditions and after block of *I*_f_ by 2 mM Cs^+^, a relatively specific blocker of *I*_f_ (Fig. 4i)—see Supplementary Discussion. Cs^+^ decreased the intrinsic heart rate of both control and heart failure mice, but the percentage decrease was significantly less in heart failure mice (Fig. 4j), consistent with a downregulation of *I*_f_ in heart failure mice. Most importantly, the intrinsic heart rate of the heart failure mice was no longer lower than that of the control mice after block of *I*_f_ by Cs^+^ (Fig. 4i).

**Figure 5:**
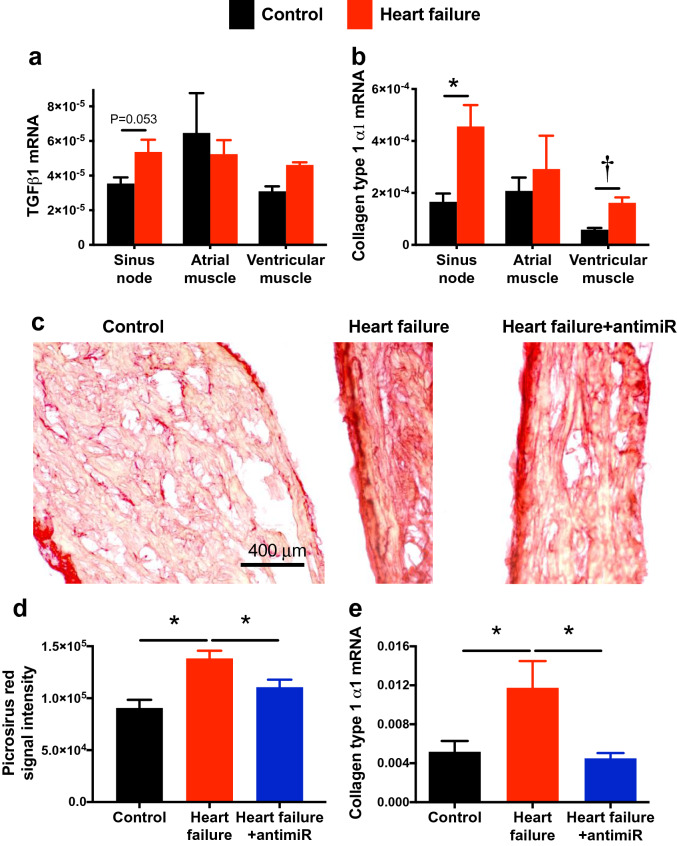
Silencing miR-370-3p improves fibrosis of the sinus node in heart failure. (**a**) Expression of mRNA for TGFβ1 in the sinus node, atrial muscle and ventricular muscle of control and heart failure mice (n = 7, 9, 6, 9, 6 and 9). One-way ANOVA revealed no significant differences; difference between the sinus node of control and heart failure mice tested using Student’s unpaired t test; P value given. (**b**) Expression of mRNA for collagen type 1 α1 in the sinus node, atrial muscle and ventricular muscle of control and heart failure mice (n = 7, 10, 7, 10, 6 and 10). *P < 0.05; Kruskal–Wallis test followed by Dunn’s multiple comparisons test. ^†^*P* < 0.05; Student’s unpaired t test. (**c**) Images of picrosirius red stained tissue sections (all at same scale) through the sinus node of a control mouse given PBS, a heart failure mouse given PBS and a heart failure mouse given antimiR-370-3p. Red stain, collagen; yellow stain, cardiac myocytes. (**d**) Mean intensity of picrosirius red staining of collagen (red signal) in the three groups of mice (n = 29, 35 and 47 sections from n = 4, 3 and 3 mice). *P < 0.05; one-way ANOVA followed by Tukey’s multiple comparisons test. (**e**) Expression of collagen type 1 α1 mRNA in the three groups of mice (n = 7, 8 and 8). *P < 0.05; one-way ANOVA followed by Tukey’s multiple comparisons test.

**Figure 6:**
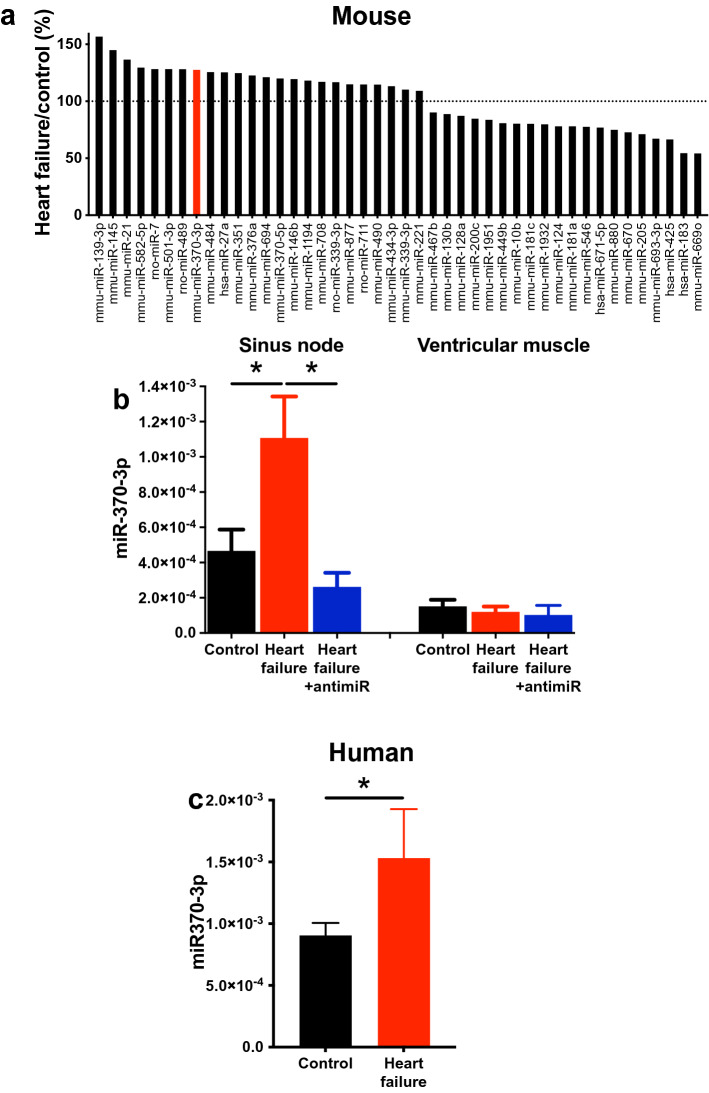
Dysregulation of microRNAs in heart failure. (**a**) 44 microRNAs that changed significantly in the sinus node in heart failure. The expression of each microRNA in heart failure mice is shown as a percentage of expression in control mice. The dotted line shows the control level. Based on data from 8 control and 10 heart failure mice. miR-370-3p is highlighted. (**b**) Expression of miR-370-3p in the sinus node (n = 7, 7 and 8) and ventricular muscle (n = 8, 9 and 7) of control mice given PBS, heart failure mice given PBS and heart failure mice given antimiR-370-3p. *P < 0.05; one-way ANOVA followed by Tukey’s multiple comparisons test. (**c**) Expression of miR-370-3p in the sinus node of control and heart failure patients (n = 3 and 5). *P < 0.05; after adjusting for effects of other factors and normalising differences between experiments, the General Linear Model was used to evaluate differences in miR-370-3p levels between control and failing human hearts.

In the sinus node of the heart failure mice, qPCR showed that there was a trend of an upregulation of the proinflammatory cytokine, TGFβ1, as well as a significant upregulation of the major collagen isoform in the heart, collagen type 1 α1 (Fig. 5a, b). This suggests there was fibrosis of the sinus node in the heart failure mice and, therefore, tissue sections through the sinus node were stained with picrosirius red (Fig. 5c), which stains collagen 1 and 3 fibres red. Image analysis showed that there was significantly more picrosirius red staining in the sinus node of the heart failure mice (Fig. 5d). This demonstrates that there was fibrosis of the sinus node in heart failure; a similar fibrosis was also observed in the atrial and ventricular muscle (Supplementary Figs. 7 and 8). The fibrosis was, therefore, not restricted to the sinus node. Both immunohistochemistry (immunolabelling of the cell membrane) and patch clamp experiments (measurement of cell capacitance) showed that there was a significant increase in the size of sinus node cells in the heart failure mice (Supplementary Fig. 9), demonstrating that there was hypertrophy of the sinus node as well as the rest of the heart (Figs. 1, 2).

**Figure 7:**
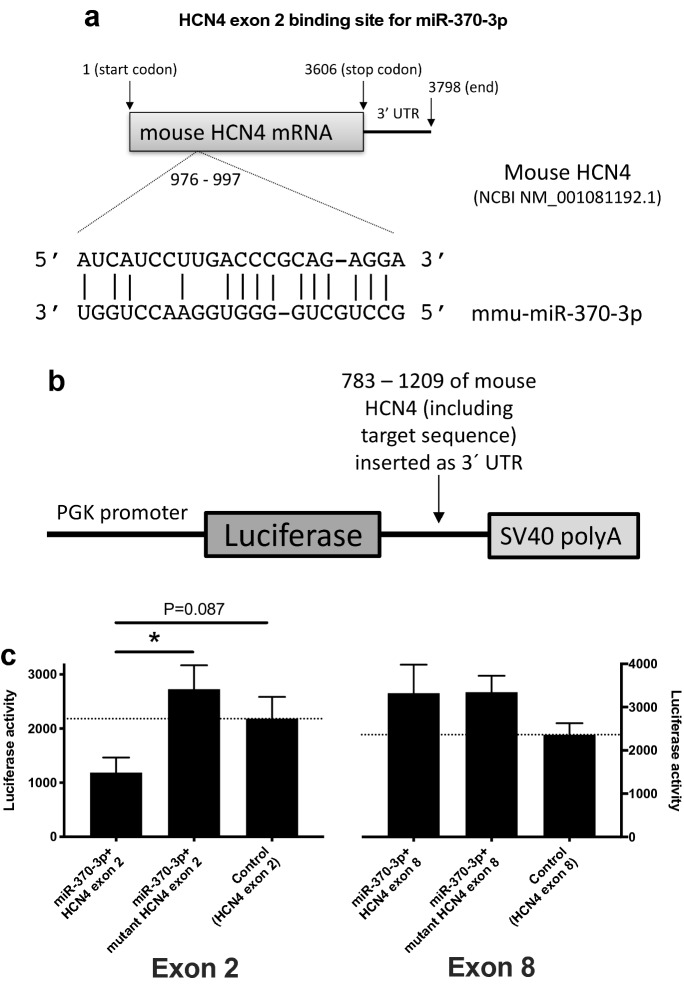
miR-370-3p regulates HCN4. (**a**) Predicted miR-370-3p binding site in exon 2 of HCN4. In the mutant, the predicted exon 2 binding site was mutated from AUCAUCCUUG**ACC**CGCAGAGGA (wild-type) to AUCAUCCUUG**CAA**CGCAGAGGA (mutant)—the corresponding nucleotides are in bold. (**b**) Luciferase reporter gene construct. PGK, phosphoglycerate kinase 1. (**c**) Luciferase reporter assay showing effect of miR-370-3p on HCN4. Effects mediated by potential binding sites in exons 2 (left) and 8 (right) were investigated. Bars show luciferase activity (surrogate of HCN4) when: miR-370-3p was incubated with the wild-type exon; miR-370-3p was incubated with a mutant exon (potential binding site mutated); and wild-type exon was not exposed to miR-370-3p. Luciferase activity is shown 24 h after co-transfection. Exon 2, n = 14, 10 and 5 measurements; exon 8, n = 7, 6 and 7 measurements. Exon 2: *P < 0.05, Kruskal–Wallis test followed by Dunn’s multiple comparisons test; in addition, the Mann–Whitney test was used to test the difference between miR-370-3p + HCN4 exon 2 and control (HCN4 exon 2 only) and the P value is shown. Exon 8: one-way ANOVA revealed no significant differences.

**Figure 8:**
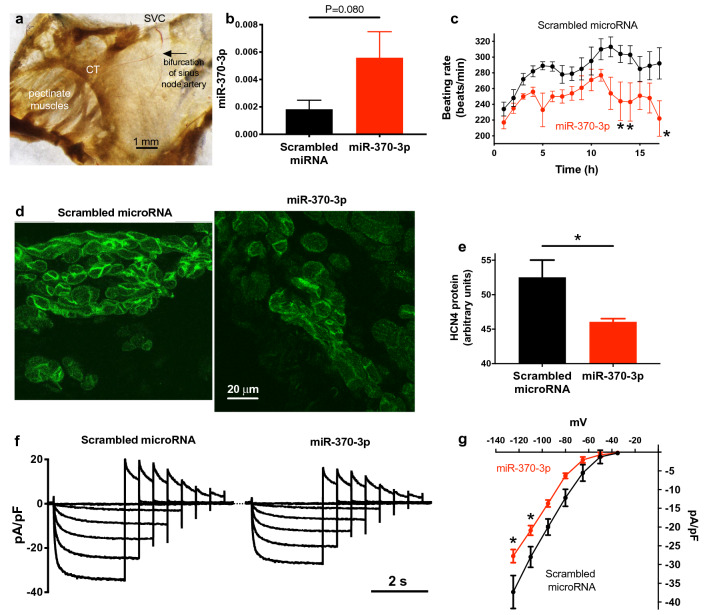
miR-370-3p regulates HCN4 and *I*_*f*_ density. (**a**) Isolated sinus node preparation showing the bifurcation of the sinus node artery where microRNA was injected. CT, crista terminalis; SVC, superior vena cava. (**b**) Expression of miR-370-3p 28–42 h after the injection of isolated sinus node preparations with a scrambled microRNA or miR-370-3p (n = 9 and 9). Data analysed using Student’s unpaired t test and P value given. (**c**) Time course of the beating rate of isolated sinus node preparations following the injection of a scrambled microRNA or miR-370-3p (n = 9 and 9). *P < 0.05; two-way ANOVA followed by Sidak’s multiple comparisons test. (**d**) Immunolabelling of HCN4 in tissue sections (shown at same scale) through the sinus node of isolated sinus node preparations that had been injected with a scrambled microRNA or miR-370-3p 28–42 h previously. (**e**) Mean intensity of immunolabelling of HCN4 in the sinus node of isolated sinus node preparations that had been injected with a scrambled microRNA or miR-370-3p 28–42 h previously (n = 2 and 3). (**f**) Families of recordings of *I*_f_ from sinus node cells that had been incubated with a scrambled microRNA or miR-370-3p for 6 h. (**g**) Mean current–voltage relationships for *I*_f_ from sinus node cells that had been incubated with a scrambled microRNA or miR-370-3p for 6 h (n = 4 and 14). *P < 0.05; two-way ANOVA followed by Sidak’s multiple comparisons test.

**Figure 9:**
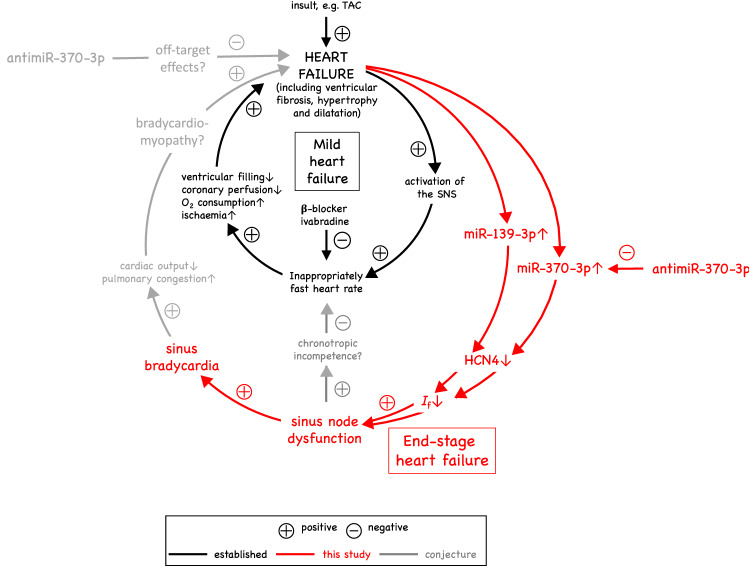
Working hypothesis: relationship between heart failure and sinus node dysfunction. The inner loop (in black) is well known. Treatment of heart failure patients with β-blockers or ivabradine reduces the heart rate, which is considered beneficial. The outer loop (in red) explains the data in this study. Conjecture as to why antimiR-370-3p should lessen heart failure and improve mortality in the mouse is shown in grey: either it has off-target effects, which lessen heart failure etc., or by improving sinus node function, it lessens heart failure etc. (possibly by relieving chronotropic incompetence and bradycardiomyopathy).

### Mechanisms underlying the remodelling of the sinus node in heart failure

What is responsible for the remodelling of the sinus node, in particular of HCN4, in heart failure? We investigated the role of transcriptional regulators (transcription factors) and post-transcriptional regulators (microRNAs) in defining the level of HCN4. HCN4 mRNA levels are regulated by the transcription factors AP1^39^, Isl1^40,41^, Mef2c^39^, Nkx2.5^42^, NRSF^43^, Tbx3^44^ and Tbx18^45^; BMP2, BMP4 and Shox2 are sinus node specific transcription factors^46^. For example, the transcription factors, Tbx3 and Tbx18, are known to control the pacemaking phenotype of the sinus node and ectopic expression of Tbx3 and Tbx18 in working myocardium causes an upregulation of HCN4 and a switch to a sinus node phenotype^44,45^. In the heart failure mice, qPCR showed no significant change in Bmp2, Bmp4, Nkx2.5, Shox2 and Tbx3 in the sinus node, but there was a significant downregulation of Tbx18 and Isl1 and upregulation of AP1, Mef2c and NRSF mRNA (Supplementary Fig. 4).

Expression of 754 rodent microRNAs in the sinus node was determined using a TaqMan Gene Expression Assay (ThermoFischer Scientific). Among the 754 microRNAs, 44 microRNAs showed a significant difference between control and heart failure mice (Fig. 6a). 24 microRNAs were significantly upregulated in the sinus node of the heart failure mice and 20 microRNAs were significantly downregulated (Fig. 6a). Of the upregulated microRNAs, four (miR-139-3p, miR-145-5p, miR-351-3p and miR-370-3p) were identified by Ingenuity Pathway Analysis software (Qiagen) as potentially targeting HCN4. RNA22 software predicted that HCN4 has 10 potential binding sites (two of which are significant) for miR-370-3p, five (two of which are significant) for miR-139-3p, two for miR-351-3p and just one for miR-145-5p (Supplementary Table 2). miR-370-3p was the subject of further study, although some experiments were also conducted on miR-139-3p (the two microRNAs with the greatest number of predicted binding sites). We focussed our investigations on the most common isoform of miR-370-3p, as defined by over 70 small RNA deep sequencing datasets in both mouse and human catalogued by the miRBase database—see Supplementary Discussion for further information. An independent qPCR assay confirmed that miR-370-3p was significantly upregulated in the sinus node in heart failure (Fig. 6b). miR-370-3p was also expressed in atrial muscle; there was a trend of upregulation in heart failure, but it was not significant (Supplementary Fig. 10). Expression of miR-370-3p in ventricular muscle was significantly less than in the sinus node and, furthermore, it did not respond to heart failure (Fig. 6b). MicroRNAs can be generated by cardiomyocytes or associated fibroblasts^47^ and, to distinguish between the two sources, expression of miR-370-3p and HCN4 as a reference was measured in isolated intact sinus node tissue and pure fibroblast populations isolated from the sinus node (Supplementary Fig. 11). Expression levels of the transcripts were normalised to RNU6. As expected, HCN4 was present in the sinus node, but absent from fibroblasts (Supplementary Fig. 11). miR-370-3p showed the same pattern (Supplementary Fig. 11) and this suggests that most miR-370-3p originates from sinus node cardiomyocytes.

miR-370 overlaps an intron in a complex set of alternately spliced transcripts from a lincRNA locus (Rian-210 and Rian-216) in mouse. The same transcripts also appear to express the rodent-specific microRNAs miR-341 and miR-1188. These three microRNAs may therefore be co-regulated in mouse. In both mouse and human, miR-139 is located in an intron of the protein-coding gene Pde2a (phosphodiesterase 2A). Therefore, it is possible that miR-139-3p and Pde2a are co-regulated, as has been shown previously for other intronic microRNAs and their host genes^48,49^. It is interesting that upregulation of miR-341, miR-1188 and Pde2a have all been linked to cardiac hypertrophy or heart failure^50–52^. Although no change in miR-1188-3p was observed in the present study (data not shown; miR-341 not investigated), qPCR showed evidence of upregulation of Pde2a mRNA in the sinus node, but not ventricle, in heart failure (Supplementary Fig. 12a).

### miR-370-3p is also upregulated in the human sinus node in heart failure

The sequence of miR-370-3p is perfectly conserved across many species including human, rat and mouse (Supplementary Fig. 13; the same is true of miR-139-3p—Supplementary Fig. 14). Experiments were conducted on the healthy sinus node from three human subjects and the sinus node from five heart failure patients to determine whether miR-370-3p is also upregulated in the human sinus node in heart failure (details of human subjects given in Supplementary Table 3). There was an upregulation of miR-370-3p in the heart failure group (Fig. 6c). The General Linear Model was used to evaluate the statistical significance of the difference in miR-370-3p between hearts with and without heart failure. After adjusting for other factors, including gender, age, heart weight and diabetes mellitus status, the effect of heart failure was statistically significant (*P* < 0.001).

### HCN4 is a target of miR-370-3p (and miR-139-3p)

The two significant potential binding sites for miR-370-3p within mouse HCN4 mRNA are in the mRNA corresponding to exons 2 and 8 and are shown in Fig. 7a (exon 2 site) and Supplementary Fig. 15 (exon 8 site). The predicted binding sites for miR-370-3p are in the coding region—recent evidence suggests that microRNA target sites in the coding region may be just as important as those in 3′-UTR^53^. Luciferase reporter gene assays were carried out to test whether the predicted binding sites are functionally important: mRNA corresponding to exon 2 (or exon 8) was inserted as the 3′-UTR of the luc2 firefly luciferase gene (constitutively active under the control of the phosphoglycerate kinase 1 promoter; Fig. 7b). The plasmid was transfected into H9C2 cells with or without plasmid expressing precursor miR-370. miR-370-3p significantly reduced luciferase activity (luciferase bioluminescence normalised to Renilla bioluminescence; surrogate of HCN4) with exon 2 of HCN4, but not with exon 8 of HCN4 (Fig. 7c). Mutation of the predicted miR-370-3p binding sequence in exon 2 abolished the effect of miR-370-3p on luciferase activity; mutation of the potential binding sequence in exon 8 had no effect (Fig. 7c). This finding demonstrates a specific interaction between miR-370-3p and HCN4, and the predicted recognition element identified in the exon 2 of HCN4 contributes to this.

The predicted binding sites for miR-139-3p were not investigated. However, using a luciferase reporter gene assay with the 3′-UTR of HCN4 cloned downstream of the luciferase gene, we have previously screened various microRNAs, including miR-139-3p, for effects mediated by the 3′-UTR of HCN4^54^. miR-139-3p significantly decreased luciferase activity (Supplementary Fig. 12b); this demonstrates a specific interaction between miR-139-3p and HCN4.

Ingenuity Pathway Analysis identified other ion channels as potential targets of miR-370-3p, but none were significantly changed at the transcript level during heart failure (Supplementary Fig. 16). However, it is known that microRNAs can operate at the level of protein translation without producing corresponding changes in steady state mRNA levels^55^ and this possibility cannot be excluded.

### Overexpression of miR-370-3p (and miR-139-3p) induces bradycardia

To test whether overexpression of miR-370-3p can produce bradycardia, we transfected isolated sinus node preparations (Fig. 8a) with miR-370-3p. 1–2 μl of 10 nM double-stranded synthetic miR-370-3p (together with Lipofectamine Transfection Reagent) was injected into the centre of the sinus node at the bifurcation of the sinus node artery using a 35G needle (Fig. 8a). This series of experiments was conducted on sinus node preparations isolated from the rat, because injection into the smaller mouse preparations was not feasible. However, miR-370-3p is conserved in human, rat and mouse (Supplementary Fig. 13) and in all three species miR-370-3p is highly predicted to affect HCN4 by Ingenuity Pathway Analysis software (Qiagen). To serve as a control, other sinus node preparations were transfected with a scrambled (non-targeting) microRNA, designed to have no sequence similarity to any known microRNA sequence in the database. Injected preparations were maintained in tissue culture. qPCR showed a 207% upregulation of miR-370-3p 28–42 h after injection (*P* = 0.080; Fig. 8b). Overexpression of miR-370-3p resulted in a significant decrease in the beating rate of the sinus node preparations (in comparison to the beating rate of preparations injected with a scrambled microRNA) and this effect possibly started ~ 5 h after injection of miR-370-3p (Fig. 8c). Tissue sections cut through the sinus node 28–42 h after injection of the preparations with miR-370-3p or the scrambled microRNA were immunolabelled for HCN4 (Fig. 8d); image analysis showed a significantly lower expression of HCN4 protein in the sinus node in preparations overexpressing miR-370-3p (Fig. 8e). In another series of experiments, isolated mouse sinus node cells were incubated with 10 nM miR-370-3p or 10 nM scrambled microRNA for 4–6 h and then *I*_f_ was recorded with the patch clamp technique (Fig. 8f). Experiments were carried out on mice of the same strain (C57BL6/N) and age as the mice used to generate the heart failure model. Figure 8g shows mean current–voltage relationships for *I*_f_ density and it shows that incubation with miR-370-3p resulted in a significant decrease in the density of *I*_f_. Taken together, these data show that miR-370-3p causes a bradycardia probably by downregulating HCN4 and thus *I*_f_.

Injection of miR-139-3p into isolated sinus node preparations also resulted in a bradycardia from 8 h after injection (Supplementary Fig. 12c). 24 h after injection, immunohistochemistry showed a significant decrease in HCN4 protein expression in the sinus node (Supplementary Fig. 12d).

### Silencing of miR-370-3p in heart failure partially restores sinus node function

If the upregulation of miR-370-3p in heart failure is responsible for the sinus node dysfunction, silencing miR-370-3p should benefit the sinus node. To test this, mice were treated with antimiR-370-3p from 21 days after TAC surgery. AntimiRs are antisense oligonucleotides modified to enhance duplex stability and have been used effectively to inhibit microRNA function in vitro and in vivo^56,57^. From 21 days after TAC surgery, mice were given a daily dose of antimiR (20 mg/kg) in phosphate buffered saline (PBS) for 3 days followed by a weekly dose for 3 weeks. Two control groups (sham-operated mice and mice subjected to TAC) did not receive the antimiR—both control groups were given the vehicle (PBS) only. There were two other control groups: sham-operated mice were given antimiR or the scrambled antimiR (20 mg/kg in PBS). The mice were sacrificed ≤ 60 days after surgery. AntimiR-370-3p injection decreased miR-370-3p levels in the sinus node of heart failure mice as compared to heart failure mice which only received an injection of PBS; the level was decreased to below that in the sham-operated mice (Fig. 6b). AntimiR-370-3p had many general beneficial effects for the heart failure mice. The most striking was that survival of the heart failure mice was significantly improved (*P* = 0.013, log-rank test): 15 out of 15 sham-operated mice survived, 17 out of 20 heart failure mice which received antimiR-370-3p survived, whereas only 6 out of 17 heart failure mice which received PBS only survived (Fig. 1a). AntimiR-370-3p restored body weight to control values (Fig. 1b, c). However, heart weight (Fig. 1e) and heart weight to tibia length ratio and heart weight to body weight ratio (Fig. 1f; Supplementary Fig. 1a) only showed a trend towards restoration. Echocardiography revealed improvements in cardiac function of heart failure mice after injection of antimiR-370-3p: left ventricle mass and internal diameter in diastole and systole were all significantly decreased towards normal, and left ventricle fractional shortening was increased towards normal (*P* = 0.052; Fig. 2).

In heart failure mice, antimiR-370-3p restored the heart rate measured in the unrestrained conscious mouse towards normal (Fig. 3a, b). It also restored the intrinsic beating rate of the isolated sinus node preparation (Fig. 3g) towards normal (the beating rate is no longer significantly different from the control). This is the first evidence that antimiR-370-3p protects the sinus node from the effects of heart failure. AntimiR-370-3p also reversed the first-degree heart block, i.e. prolongation of the PR interval (Fig. 3c). This suggests that antimiR-370-3p also protects the atrioventricular node from the effects of heart failure. This is not surprising, because the two nodes share much in common^29^. However, antimiR-370-3p did not reverse the effects of heart failure on the His-Purkinje system: it did not restore the QRS duration (Fig. 3d). However, antimiR-370-3p did restore the QT and corrected QT intervals to normal (Fig. 3e, f), which is another indicator that the severity of heart failure was reduced. There was other evidence that antimiR-370-3p protected the sinus node from the effects of heart failure. AntimiR-370-3p significantly restored HCN4 mRNA beyond control (Fig. 4d), HCN4 protein to control (Fig. 4e, f), and *I*_f_ density towards control (Fig. 4g, h). The responsiveness of the isolated sinus node to Cs^+^ block of *I*_f_ was also increased towards control (the responsiveness was no longer significantly different from the control; Fig. 4j). Why HCN4 mRNA but not the other measures should rebound beyond the control level is not known. Finally, antimiR-370-3p also partially reversed the hypertrophy of sinus node cells (Supplementary Fig. 9).

Figure 5d, e shows that antimiR-370-3p largely prevented fibrosis of the sinus node in heart failure: picrosirius red staining was significantly restored towards normal and collagen type 1 α1 mRNA was significantly restored to normal. AntimiR-370-3p also significantly reduced fibrosis in the atrial and ventricular muscle in heart failure (Supplementary Figs. 7 and 8).

In the further control experiments on sham-operated mice, the antimir had no effects (as compared to sham-operated mice receiving PBS alone) on echocardiography parameters including heart rate (Supplementary Table 4), and the scrambled antimiR had little or no effect on mortality, body weight, HCN4 expression or *I*_f_ density (Figs. 1a, c, 4f, h).

## Discussion

For the first time, this study has shown that the sinus and atrioventricular node dysfunction in heart failure can be moderated or reversed by administration of an antimiR. This has important novel clinical implications, because bradyarrhythmias (a consequence of sinus node and atrioventricular node dysfunction) are linked to ~ 50% of deaths of heart failure patients^6–8^. The CARISMA study showed that, in post-myocardial infarction (MI) patients with heart failure, sinus and atrioventricular node dysfunction (as indicated by sinus bradycardia and intermittent high-degree atrioventricular block, respectively) are associated with a very high risk of cardiac death^58^.

We have shown that in the mouse TAC model of heart failure there is hypertrophy and dilatation of the heart and a decrease in the left ventricle fractional shortening, body weight and survival as expected (Figs. 1, 2). There is also evidence of dysfunction of the sinus node (bradycardia), atrioventricular node (first-degree heart block) and His-Purkinje system (increase in QRS duration) in the heart failure model (Fig. 3) as expected—all these changes are observed in heart failure patients e.g.^13,59,60^. The data suggests that the sinus node dysfunction is the result of a downregulation of HCN4 mRNA and protein and subsequently the density of *I*_f_ (Fig. 4). There was no evidence of involvement of the Ca^2+^ clock in heart failure: there were no significant changes in Ca^2+^ clock transcripts in the sinus node in heart failure and no significant changes in selected Ca^2+^ clock transcripts following antimiR-370-3p (Supplementary Fig. 5). Verkerk et al.^34^ recently concluded that Ca^2+^ cycling properties are conserved despite bradycardic effects of heart failure in sinus node cells (rabbit model of pressure and volume overload studied). On the other hand, Shinohara et al.^61^ found that heart failure (induced by rapid ventricular pacing) results in Ca^2+^ clock malfunction in the sinus node of dogs. There was measurable fibrosis in the sinus node in heart failure (Fig. 5) consistent with other work^14^. Increased extracellular matrix deposition within the sinus node has been shown to disrupt electrical coupling between nodal cells that can result in slowed impulse propagation, sinus node exit block and sinus pauses^62^. The possibility that the observed increase in fibrosis plays a role in sinus node dysfunction in heart failure cannot be excluded, although, in the isolated sinus node, injection of miR-370-3p perhaps began to slow pacemaking after 5 h (Fig. 8c) and this is likely to be too short for significant fibrosis.

We have shown a widespread dysregulation of transcription factors and microRNAs in the sinus node in heart failure (Supplementary Fig. 4; Fig. 6). Of the transcription factors known or predicted to regulate HCN4 (AP1, Isl1, Mef2c, Nkx2.5, NRSF, Tbx3 and Tbx18), two (Nkx2.5 and Tbx3) did not change in the sinus node in heart failure (Supplementary Fig. 4). HCN4 is a direct transcriptional target of AP1 and Mef2c^39^, but these transcription factors were significantly upregulated in the sinus node in heart failure (Supplementary Fig. 4) and cannot explain the downregulation of HCN4. However, NRSF is potentially involved: NRSF was significantly upregulated in the sinus node in heart failure (Supplementary Fig. 4); NRSF is a transcriptional repressor and binds to a silencer element within an intron of HCN4 and prevents its transcription^43^. Isl1 and Tbx18 are also potentially involved: both Isl1 and Tbx18 were significantly downregulated in the sinus node in heart failure (Supplementary Fig. 4) and downregulation of Isl1 has been shown to downregulate HCN4^40,41^ and Tbx18 has been shown to upregulate HCN4^45^; however, Tbx18 is a transcriptional repressor^63^ and is unlikely to affect HCN4 directly. We observed significant changes in 44 microRNAs (Fig. 6), of which five potentially target HCN4, although only four of these were upregulated—appropriate to explain the downregulation of HCN4 (Supplementary Table 2). Of these four, miR-370-3p and miR-139-3p had the greatest number of potential binding sites on HCN4 (Supplementary Table 2). Using a luciferase reporter assay, both microRNAs were shown to be capable of regulating HCN4 (Fig. 7 and Supplementary Fig. 12b). Both microRNAs, when overexpressed in the isolated sinus node, downregulated HCN4 and slowed sinus node pacemaking (Fig. 8c–e and Supplementary Fig. 12c, d). In single sinus node cells, miR-370-3p was also shown to downregulate *I*_f_ density (Fig. 8f, g). In summary, we have identified five factors that could be involved in the downregulation of HCN4 in the sinus node in heart failure: Isl1, NRSF, Tbx18, miR-139-3p and miR-370-3p.

miR-370-3p was the subject of further study. In the sinus node in heart failure, silencing of miR-370-3p by antimiR-370-3p blunted or reversed the downregulation of HCN4 mRNA and protein and the density of *I*_f_; antimiR-370-3p also blunted the bradycardia in vivo (Figs. 3, 4). These data suggest that antimiR-370-3p protects the sinus node in heart failure. AntimiR-370-3p also reversed the prolongation of the PR interval in heart failure (Fig. 3c) and this suggests that antimiR-370-3p also protects the atrioventricular node in heart failure; this is not surprising, because the sinus and atrioventricular nodes are similar in terms of embryology, ion channel expression and electrophysiology^29^. Heart failure resulted in a prolongation of the QRS complex (Fig. 3d), characteristic of heart failure patients^29^. We attribute this to slowed conduction through the His-Purkinje system, although theoretically it could be the result of fibrosis within the ventricles. However, antimiR-370-3p reversed ventricular fibrosis in heart failure (Supplementary Fig. 8), but it did not restore the QRS duration to baseline values (Fig. 3d), and this suggest that miR-370-3p-independent electrical remodelling within the His-Purkinje system may be the dominant underlying mechanism of QRS prolongation in this model.

### Other actions of antimiR-370-3p

AntimiR-370-3p also reversed fibrosis in the sinus node (Fig. 5), atrial muscle (Supplementary Fig. 7) and ventricular muscle (Supplementary Fig. 8) in heart failure. However, this is unlikely to be a direct action of antimiR-370-3p. First, although miR-370-3p is expressed in atrial muscle, it was not significantly upregulated in heart failure (Supplementary Fig. 10), and miR-370-3p is poorly expressed in ventricular muscle where it was unresponsive to heart failure and antimiR-370-3p (Fig. 6b). Secondly, miR-370 has been reported to be anti-fibrotic in heart^64^ and liver^65^. In relation to fibrosis and hypertrophy in angiotensin II-induced cardiac hypertrophy, Bang et al.^66^ demonstrated that microRNA can be secreted by cardiac fibroblasts in exosomes and exert a paracrine action on cardiac myocytes. However, Yuan and Gao^64^ showed that exposure of cultured neonatal rat cardiac fibroblasts to angiotensin II for 24 h resulted in a downregulation of miR-370 (as well as an upregulation of TGFβ1 and TGFβRII). Yuan and Gao^64^ showed that TGFβRII is a target of miR-370 and concluded miR-370 is anti-fibrotic. The experiment shown in Supplementary Fig. 11 suggests that the majority of miR-370-3p is expressed in the cardiomyocytes of the sinus node.

Antimir-370-3p also partially reversed hypertrophy of sinus node cells (Supplementary Fig. 9) and ventricular hypertrophy and dilatation (Figs. 1, 2). However, again this is unlikely to be a direct action of antimiR-370-3p. Although miR-370-3p potentially targets transcripts linked to cardiac hypertrophy (Supplementary Table 5), once again the problem is the poor expression of miR-370-3p in ventricular muscle and its unresponsiveness to heart failure (Fig. 6b). Finally, antimiR-370-3p partially rescued ventricular function, largely prevented the loss of body weight and reduced mortality (Figs. 1, 2). These are likely to be indirect actions of antimiR-370-3p: the improvement in ventricular function is likely to be a consequence of the improvement in ventricular fibrosis and hypertrophy, and the improvement in body weight and mortality is likely to be a consequence of the improvement in ventricular function. If the improvement in ventricular fibrosis and hypertrophy are not the result of a direct action of antimiR-370-3p on the ventricles, what is responsible? One possibility is that the improvement in ventricular fibrosis and hypertrophy is an indirect consequence of an unknown action of antimiR-370-3p on another organ (an off-target effect). For example, miR-370-3p has been previously reported to control lipid metabolism and to lead to an accumulation of triglycerides in the liver^67^.

### Working hypothesis

Figure 9 summarises events in heart failure (well established in black, established by this study in red and conjectural in grey). It is well established that in mild heart failure (as shown by the inner loop in black in Fig. 9), there is activation of the sympathetic nervous system resulting in an increase in heart rate^2,68^. This is despite the fact that there may be some underlying dysfunction of the sinus node and a decrease in the intrinsic heart rate^2,9,13^. There is consensus that a high heart rate will worsen heart failure^4^, which is a reason why the cardiologist will reduce the heart rate by prescribing a β-blocker or ivabradine^4,5^. However, in end-stage heart failure (as shown by the part of the outer loop in red in Fig. 9), we propose the sinus node dysfunction becomes more severe and the sinus node can no longer be supported by activation of the sympathetic nervous system. This may result in bradycardia in the mouse (Fig. 3) and also in the human: in the CARSIMA study, the heart rhythm of 297 post-MI heart failure (ejection fraction < 40%) patients were monitored over two years using implantable loop recorders and there was a 7% incidence of sinus bradycardia (≤ 30 beats/min lasting ≥ 8 s) and a 5% incidence of sinus arrest (≥ 5 s) as well as a 10% incidence of high-degree atrioventricular block (≤ 30 beats/min lasting 8 s)^58^. The sinus node dysfunction may also be responsible for chronotropic incompetence (inability of the heart rate to increase commensurate with increased activity, well known in heart failure^2,69^). The prevalence of chronotropic incompetence in chronic heart failure is high, approaching 70% of patients^70^. While the mechanism of chronotropic incompetence has previously been attributed to downregulation of beta-receptors and desensitization in the presence of increased circulating catecholamine levels^71–73^, more recent evidence suggests that reduced sinus node reserve (i.e. sinus node dysfunction) may be responsible^13,69^. The possible link of chronotropic incompetence to sinus node dysfunction is shown in grey (i.e. as conjectural) in Fig. 9. This study shows that the sinus node dysfunction (and perhaps the atrioventricular node dysfunction) is the result of an upregulation of miR-370-3p (and perhaps miR-139-3p), downregulation of HCN4 and downregulation of *I*_f_ (Fig. 9). We propose that the nodal dysfunction in heart failure should not be ignored and instead be treated and perhaps an antimiR offers a suitable means as shown by this proof-of-principle study.

The cause of the upregulation of miR-370-3p (and perhaps miR-139-3p) is outside the scope of this study. However, the effect of the beta blocker, bisoprolol, in heart failure on the sinus node has been investigated by Du et al.^74^ in the rat and, interestingly, it partially reverses the sinus bradycardia and sinus node dysfunction and it fully reverses the downregulation of HCN4. This suggests that the remodelling may be due to the hyperadrenergic state seen in chronic heart failure^74^.

The partial rescue of ventricular function, body weight and mortality by antimiR-370-3p (Figs. 1, 2) could be an off-target effect (shown in grey, i.e. as conjectural, in Fig. 9) as explained above. However, an alternative explanation is that the sinus (and atrioventricular) node dysfunction in heart failure worsens heart failure by causing a ‘bradycardiomyopathy’ (again shown in grey, i.e. as conjectural, in Fig. 9). It has been shown that the development of bradycardia can lead to new symptoms of heart failure and decompensation of pre-existing heart failure^75–84^. The effect of heart rate can be *tentatively* estimated: during heart failure, the mean heart rate decreased from ~ 795 to ~ 681 beats/min (Fig. 3a) and the mean stroke volume decreased from 71.1 to 55.5 μl (Supplementary Table 1; this assumes that the stroke volume in the conscious mouse behaves in the same manner as the stroke volume in the anaesthetised mouse). The decrease of the stroke volume alone will decrease cardiac output by ~ 22%, whereas the decrease of heart rate alone will decrease cardiac output by ~ 14%; the effect of heart rate is, therefore, tentatively predicted to be considerable. In addition, PR prolongation (causing atrioventricular delay) is associated with diastolic mitral regurgitation and decreased stroke volume^85,86^. In this scenario, antimiR-370-3p, by relieving sinus node dysfunction (and also atrioventricular node dysfunction) in heart failure, will indirectly improve ventricular fibrosis, hypertrophy, dilatation and function and, consequently, body weight and mortality. It is perhaps pertinent that antimiR-370-3p failed to improve the stroke volume (Supplementary Fig. 1). Therefore, any improvement in cardiac output would have been the result of the improvement in the heart rate (Fig. 3a, b).

## Methods

### Heart failure model

Care and use of laboratory animals conformed to the UK Animals (Scientific Procedures) Act 1986 and Directive 2010/63/EU of the European Parliament. Ethical approval for all experimental procedures was granted by the University of Manchester Animal Welfare and Ethical Review body. Male mice (C56BL/6N strain) of 8–9 weeks of age (25–30 g) were used. All TAC procedures were carried out as described previously^87^ and performed by the same experienced operator who was blinded to animal details. The animals were anaesthetised with inhalation isoflurane (2–3%). The mice were ventilated with a tidal volume of 0.13 ml and a respiratory rate of 110 breaths per minute (Harvard Apparatus). A longitudinal incision of 2–3 mm was made in the proximal sternum to allow visualisation of the aortic arch. The transverse aortic arch was ligated with three knots using a 7–0 prolene suture between the innominate and left common carotid arteries with an overlaid 27-gauge needle. The needle was then removed, leaving a discrete region of constriction to maintain a pressure gradient of about 35–50 mm Hg between the left and right carotid arteries. For the sham procedure, the aortic arch was isolated and entwined with a 7–0 prolene suture without ligation. Heart rate and body weight of the mice were measured approximately every week. Mice were sacrificed by cervical dislocation when they showed symptoms of heart failure (loss of the normal lustre of the fur, rapid breathing, weakness, lack of movement and, most importantly, loss of more than 20% of body weight).

### AntimiR administration

AntimiR-370-3p (targeting the short form of miR-370-3p; product code 199,900), was purchased from Exiqon. Exiqon antimiRs use locked nucleic acid technology and have been widely used in other studies e.g. ^88^. AntimiR-370-3p was diluted with sterile PBS (catalogue no. J611196, Alfa Aesar) following the recommendations of the manufacturer. All solutions were mixed by vortexing for 10 s and incubated for at least 15 min at 37 °C before injection. Each mouse received antimiR (20 mg kg^−1^) through intraperitoneal injection consecutively for three days (3 weeks after TAC surgery) and three additional injections were performed once a week for the following 3 weeks; a similar dosing procedure has been used previously^89^. Other mice received an injection of PBS without added antimiR. All injections were carried out using a 30-gauge needle syringe.

### ECG recording from conscious mice

ECGs were recorded non-invasively in mice using the ECGenie recording enclosure (Mouse Specifics). The ECGenie system comprises a platform with embedded paw-sized electrodes connected to an amplifier (e-MOUSE). Signals were collected and analysed using PowerLab and LabChart (ADInstruments, version 7). Each mouse was acclimatised to the setup for 10 min before data collection. Only data from continuous recordings were used in the analysis. A peak detection algorithm on LabChart enabled R-wave identification using Fourier analysis and linear time-invariant digital filtering of frequencies below 3 Hz and above 100 Hz to minimise environmental signal disturbances. An example of the use of the algorithm is shown in Supplementary Fig. 17. The heart rate of healthy conscious mice measured using the ECGenie system in this study was ~ 760 beats/min (Fig. 3a). This is within the range of reported heart rates in mice (310–840 beats/min; https://web.jhu.edu/animalcare/procedures/mouse.html).

### ECG recording from anaesthetised mice

Anaesthesia was induced in mice with 2% isoflurane (Isoflo, Portugal) in 100% O_2_ with a delivery rate of 2 l min^−1^ until loss of the righting reflex. After induction, the animals were moved to a homoeothermic blanket and placed in dorsal recumbence. Anaesthesia was then maintained with isoflurane in 100% O_2_ with a flow of 1.5 l min^−1^ administered by means of a facemask connected to a coaxial circuit (Fluovac anaesthetic mask, Harvard Apparatus). The ECG was recorded using electrodes attached to the two front paws and the back right paw (looking at the belly of the mouse). RR, PR, QT and corrected QT intervals and QRS duration were recorded using a PowerLab/4SP with a ML135 Dual Bio amplifier (ADInstruments).

### Echocardiography on anaesthetised mice

Blinded to animal details, transthoracic echocardiography was performed on anaesthetised mice using an Acuson Sequoia C256 system (Siemens) and a 14-MHz probe. Mice were lightly anaesthetised with ~ 1% isoflurane, maintaining the heart rate at ~ 450 beats/min. M-mode parasternal short-axis views were taken to determine left ventricle internal dimension in diastole (LVIDd) and systole (LVIDs), posterior wall thickness in diastole (LVPWd) and systole (LVPWs), and interventricular septum thickness in diastole (IVSd) and systole (IVSs) over three cardiac cycles; all measurements were in mm. Left ventricle fractional shortening (%) was calculated as ((LVIDd − LVIDs)/(LVIDd)) × 100. Left ventricle mass (mg) was calculated^90^ as 1.05 × ((LVIDd + LVPWd + IVSd)^3^ − (LVIDd)^3^).

### Tissue electrophysiology

The beating rate of the isolated sinus node was determined by extracellular potential recording as described previously^91,92^. After sacrifice of a mouse by cervical dislocation, the heart was rapidly removed and placed in Tyrode’s solution containing (in mM): 100 NaCl, 4 KCl, 1.2 MgSO_4_, 1.2 KH_2_PO_4_, 1.8 CaCl_2_, 25 NaHCO_3_ and 10 glucose. The solution was bubbled with 95% O_2_ and 5% CO_2_ to give a pH of 7.4. A right atrial preparation encompassing the sinus node and including the superior vena cava was rapidly dissected and superfused with warm (37 °C) Tyrode’s solution at a rate of 10 ml min^−1^. Extracellular potentials were recorded from the sinus node preparation using two stainless steel pin electrodes (as well as an earth electrode). These recording electrodes interfaced with a Neurolog system (Digitimer) with low-pass and high-pass filters adjusted to optimise the signal-to-noise ratio. Extracellular potentials were continuously recorded using a PowerLab and LabChart version 7 software (ADInstruments). After dissection, the preparation was allowed to stabilise for 30 min and then the mean heart rate was calculated over 500 beats. The effect of 2 mM CsCl on the beating rate was studied: the superfusing solution was changed to Tyrode’s solution containing CsCl and, after 25 min of treatment, the heart rate was recorded for 5 min. The preparation was then washed with normal Tyrode’s solution for 30 min, during which the beating rate approached baseline values.

### Sinus node cell isolation and patch clamp

After sacrifice of a mouse, the heart was removed and kept in cold Tyrode’s solution during transport. The sinus node was then dissected and cut into 5–6 strips and then small square-shaped tissue pieces. The nodal tissues were dissociated into single cells by a standard enzymatic and mechanical procedure^93^. The tissues were suspended at 35 °C in low Ca^2+^/Mg^2+^ solution containing (in mM): 140 NaCl, 5.4 KCl, 0.5 MgCl_2_, 1.2 KH_2_PO_4_, 50 taurine, 5.5 glucose, 5 HEPES–NaOH, pH = 6.9 with NaOH. Next the tissues were incubated for 30 min with enzyme solution containing 200 μM CaCl_2_, collagenase IV (224 U ml^−1^, Worthington), elastase (1.42 U ml^−1^, Sigma-Aldrich) and protease (0.45 U ml^−1^, Sigma-Aldrich) dissolved in low Ca^2+^/Mg^2+^ solution. Tissues were washed and incubated for a further 10 min with KB solution containing (in mM): 70 L-glutamic acid, 10 DL-beta-hydroxybutyric acid, 10 KH_2_PO_4_, 10 taurine, 0.1 EGTA-KOH, 80 KOH, 20 KCl, 10 HEPES–KOH, pH = 7.4 with KOH. Isolated sinus node cells were stored at 4 °C for the day of the experiment in Tyrode’s solution containing (in mM): 140 NaCl, 5.4 KCl, 1.8 CaCl_2_, 1 MgCl_2_, 5.5 glucose, 5 HEPES–NaOH, pH = 7.4 with NaOH. The cells were incubated at 4 °C for 4–6 h with 10 nM miR-370-3p or scrambled microRNA in Tyrode’s solution. Funny current, *I*_f_, was recorded from spindle-shaped or elongated-spindle shaped cells using a patch electrode in the whole-cell configuration during superfusion of Tyrode’s solution. BaCl_2_ (1 mM) and MnCl_2_ (2 mM) were added to avoid contamination from other ionic currents. The bath temperature was 35 ± 0.5 °C. The pipette solution contained (in mM): 130 K-aspartate, 10 NaCl, 2 CaCl_2_ (pCa = 7), 2 MgCl_2_, 10 HEPES, 5 EGTA, 2 ATP(Na_2_), 0.1 GTP, 5 creatine phosphate, pH 7.2. Current was recorded during voltage steps to − 35 to − 125 mV from a holding potential of − 35 mV and normalised to cell capacitance. Data were acquired at 1 kHz using an Axopatch 200 amplifier and pClamp 8 (Molecular Devices, Sunnyvale, CA, USA). Data were analysed off-line using Clampfit 10 (Molecular Devices, Sunnyvale, CA, USA), Origin 8 (Origin Lab Corp., Northampton, MA, USA) and GraphPad Prism 6 (GraphPad Software, Inc.).

### Sinus node culture

This was carried out as described previously^94^. The sinus node was dissected in sterile conditions at 37 °C. MicroRNA mimic (mirVana miRNA Mimic; ThermoFisher Scientific) or scrambled microRNA (ThermoFisher Scientific) was mixed with Lipofectamine RNAiMAX Transfection Reagent (ThermoFisher Scientific) and injected in the sinus node preparation using a NanoFil 10 μl syringe (World Precision Instruments) at the birfurcation of the sinus node artery. The sinus node preparation was then transferred to sterile culture medium and incubated at 37 °C/5% CO_2_ for 48 h. Opti-MEM I Reduced Serum Medium (ThermoFisher Scientific) was used for the first 10 h and then Advanced DMEM/F-12 (ThermoFisher Scientific) was used for the rest of the 48 h. The isolated tissue preparation was thin (< 200 μm) and could be supported by superfusion. The culture medium used contained Advanced DMEM/F-12 (Dulbecco's Modified Eagle Medium/Ham's F-12, Life Technologies), fetal bovine serum (Life Technologies) and l-glutamine-penicillin–streptomycin (Sigma-Aldrich). Extracellular potentials were recorded from the right atrial appendage using two 0.15 mm diameter stainless steel needles (JCM Health) insulated with silicone to within 3–4 mm of the tips^46^. The electrodes were pinned into opposite sides of the preparation. The culture medium was grounded with a 0.5 mm silver wire earth electrode. The electrodes were connected to a Neurolog system (Digitimer) and filtered with suitable low and high frequency pass filters. Extracellular potentials were continuously recorded using Powerlab and Chart software (ADInstruments). The average rate was calculated via the detection of a deflection greater than two standard deviations as a paced beat.

### RNA isolation and qPCR

 ~ 1 × 1 mm sinus node tissue samples were taken from isolated preparations from the intercaval region opposite the main branch of the crista terminalis. Samples were frozen in liquid N_2_ and stored at − 80 °C until use. Atrial samples (from the right atrial appendage opposite the sinus node) and ventricular samples (from the middle of the left ventricular free wall) were treated in the same way. Total RNA was isolated from the samples using the mirVana miRNA Isolation Kit with phenol (Life Technologies) according to the manufacturer’s instructions. RNA purity and quantity was determined using a NanoDrop ND-1000 spectrophotometer (NanoDrop Technologies). Samples with an OD260/280 reading between 1.8 and 2.1 were used. First strand cDNA was synthesised using Superscript II reverse transcriptase (Invitrogen). qPCR was performed using an ABI Prism 7,900 HT Sequence Detection System (Applied Biosystems). qPCR for various transcripts was carried out using 96-well plates. The reaction mixture comprised 1 μl of cDNA, 1 × Qiagen assay, 1 × SYBR Green Master Mix (Applied Biosystems) and DNase-free water. All samples were run in triplicate. The reaction conditions were: denaturation step of 95 °C for 10 min followed by 40 cycles of amplification and quantification steps of 95 °C for 30 s, 60 °C for 30 s and 72 °C for 1 min. The melt curve conditions were: 95 °C for 15 s, 60 °C for 15 s and 95 °C for 15 s. mRNA expression normalised to the expression of the reference RNA, 18S, was calculated using the ΔCt method. Stability of three reference RNAs (18S, Ipo8 and HPRT) were evaluated using the algorithm geNorm and 18S was confirmed be the best reference RNA based on its M value (Supplementary Fig. 18).

### Reverse transcription, preamplification and qPCR for microRNAs

138 ng of total RNA from each sample was reverse-transcribed using the TaqMan microRNA Reverse Transcription Kit (Cat. No. 4366597) and the product (2.5 μl) was preamplified using Megaplex PreAmp Primers Pool A and B (which is a set of pre-defined pools of 380 stem-looped reverse transcription primers) and TaqMan PreAmp Master Mix (Applied Biosystems) in a 25 μl PCR reaction. The preamplification cycling conditions were 95 °C for 10 min, 55 °C for 2 min and 75 °C for 2 min followed by 12 cycles of 95 °C for 15 s and 60 °C for 4 min. The preamplified cDNA was diluted with 0.1 × TE (pH 8.0) to 100 μl and then 10 μl of diluted cDNA was analysed on a TaqMan Array Card run on the ABI Prism 7,900 HT Sequence Detection System. qPCR was performed using TaqMan Array Rodent MicroRNA A + B Cards Set v3.0 (ThermoFisher Scientific). The two-card set contains a total of 384 TaqMan microRNA assays per card. The set enables accurate quantitation of 754 rodent miRs. Included on each array are three TaqMan microRNA assay endogenous controls to allow data normalisation and one TaqMan microRNA assay not related to rodent as a negative control (ath-miR-159a). Results were analysed using RQ Manager (Applied Biosystems) and RealTimeStatMiner (Integromics). Average threshold cycle (Ct) values were obtained using RQ Manager, and amplification curves were analysed to check for experimental errors. Ct values were calculated using automatic baseline settings and a threshold of 0.2. Since a Ct value of 36 represents single molecule template detection, Ct values > 36 were considered to be below the detection level of the assay. Therefore, only the miRNAs with a Ct ≤ 36 were included in the analyses. RealTime StatMiner software was used for further analysis: the GeNorm stability score method was used to examine the suitability of the potential reference miRs for use individually and also in combination; U6 was used. Data were calculated as $2^{{ - \Delta C_{t} }}$, where ΔCt is ΔCt _(reference)_ − ΔCt _(target)_.

Rather than the TaqMan Array Rodent MicroRNA A + B Cards Set v3.0, in some cases miR-370-3p (short form) and miR-139-3p were measured using individual qPCR assays. A combination of U6 and RNU5G was used as the reference. The assays used and the target sequences of the assays are shown in Supplementary Table 6.

### microRNA bioinformatics

Identification of microRNAs targeting HCN4 was performed using the web-based software package Ingenuity Pathway Analysis (IPA; Ingenuity Systems). The IPA tool has a microRNA target filter function that relies on experimentally validated interactions from the TarBase database^95^, miRecords (https://mirecords.biolead.org/), TargetScan (https://www.targetscan.org/), and manually curated microRNA-related findings within the IPA knowledgebase. The latter pertains exclusively to information in the published scientific literature, whereas the first three methods rely on text mining-assisted curation (TarBase) or algorithms (TargetScan and miRecords) that find mRNA targets by searching for the presence of conserved 8mer (≥ 0.8 conserved branch length) and 7mer sites that match the seed region of each microRNA^96^. Using this approach, we identified miR-128a, miR-139-3p, miR-145-5p, miR-351-3p and miR-370-3p as potentially targeting HCN4. Predicted miR-370-3p target binding sites were obtained from RNA22 (https://cm.jefferson.edu/rna22/). Potential targets of miR-370-3p were identified using Ingenuity software with TargetScan Mouse v.5.0 prediction software (https://www.ingenuity.com/products/ipa).

### Isolation and culture of sinus node fibroblasts

A pure culture of sinus node fibroblasts was obtained based on a previously described method by Moro et al.^97^.

## Measurement of microRNAs in human sinus node

All human heart tissue research was approved by the Ohio State University Institutional Review Board and was in compliance with all relevant ethical regulations. No organs or tissues were procured from prisoners, pregnant women or children (< 18 years). Informed consent for tissue collection was obtained from transplant patients and families of donors. To prevent any potential identification, human hearts used in this study were de-identified and labelled with six-digit random codes for reference. Therefore, investigators are unable to link any specimen to any protected health information or individual. Hearts with intact sinus node pacemaker complexes from transplant patients (n = 4) and human donor hearts (n = 4) (for patient information see Supplementary Table 3) were obtained from the Ohio State University Cardiac Transplant Team or LifeLine of Ohio Organ Procurement Organization.

Human sinus node tissue was acquired by a protocol described previously^98^. Total RNA was isolated from the samples using the mirVana miRNA Isolation Kit with phenol (Life Technologies; catalogue number, AM1560) according to the manufacturer’s instructions. RNA purity and quantity was determined using a NanoDrop ND-2000 spectrophotometer (NanoDrop Technologies). Samples with an OD260/280 reading between 1.8 and 2.1 were used. 10 ng of total RNA from each sample was reverse-transcribed using the TaqMan microRNA Reverse Transcription Kit (Thermo Fisher Scientific; catalogue number, 4366596) with reverse transcription primer from each assay in a 15 μl reverse transcription reaction. The assays were purchased from Thermo Fisher Scientific, including TaqMan microRNA Assay has-miR-370 (assay ID, 002275) and TaqMan microRNA Control Assay U6 (assay ID, 001973). The reverse transcription cycling conditions were 16 °C for 30 min, 42 °C for 30 min and 85 °C for 5 min. Product (1.3 μl) from the reverse transcription reaction was amplified using TaqMan Small RNA Assay and TaqMan Universal PCR Master Mix (Thermo Fisher Scientific; catalogue number, 4324018) in a 20 μl PCR reaction. qPCR was performed using a BIO-RAD CFX Connect real-time system (Bio-Rad Laboratories) and cycling conditions were 95 °C for 10 min, followed by 40 cycles of 95 °C for 15 s and 60 °C for 1 min. Average threshold cycle (Ct) values were obtained using CFX Maestro software (Bio-Rad Laboratories) and amplification curves were analysed to check for experimental errors. U6 was used as an endogenous control. Data were calculated as 2^−ΔCt^, where − ΔCt is − (Ct_miR-370-3p_ − Ct_U6_). All samples were run in duplicate, and qPCR was repeated three times.

### Immunohistochemistry and picrosirius red staining

This was carried out as described previously^99^. Briefly, sinus node preparations were immersed in optimal cutting temperature compound (VWR) and frozen in liquid N_2_. Atrial samples (from right atrial appendage opposite sinus node) and ventricular samples (from middle of left ventricular free wall) were treated in the same way. Frozen sections (20 μm) were fixed in 10% formaldehyde, permeabilised with 0.1% Triton X-100 for 30 min, blocked with 1% bovine serum albumin and incubated with anti-HCN4 raised in rabbit (Millipore, AB5808; 1:100). Finally, sections were incubated with fluorescein isothiocyanate (FITC) (Millipore, AP182F; 1:100) or CY3 (Millipore, AP182C; 1:400) conjugated secondary antibodies raised in rabbit. Immunofluorescent labelling was carried out in batches with each batch comprising four slides from sinus node injected with microRNA-370-3p and four slides from sinus node injected with scrambled microRNA with three sections per slide. The sections were from approximately the same level in the sinus node. Images were collected on a Zeiss Imager Z1 upright confocal microscope. For HCN4 protein, high magnification images (×63) were collected using the same microscope parameters. 10–15 images per region per animal were collected. Picrosirius red staining was carried out on cryosections containing HCN4 expressing tissue and imaged using a polarizing microscope. Velocity software (Improvision, UK) was used to measure the signal intensity (in arbitrary units) for HCN4 and picrosirius red staining. Signal intensity was only measured in user-defined regions.

### Generation of luciferase reporter gene

To generate the luciferase reporter constructs the mouse HCN4 exon2 and the partial fragment of mouse HCN4 exon 8, which contained predicted binding sites of miR-370-3p, were amplified by PCR and cloned into the pmiR-GLO plasmid (Promega) as 3′-UTR sequence downstream of the luciferase gene. Primer sequence for the PCR reactions were as follows:

HCNEx2-FSac 5′ CTCAATGAGCTC CCTGCCCTCACTCTCCTG.

HCNEx2-RXho 5′ CGGAGTCTCGAGCACCTCTTCCCACTGATGA.

HCNEx8-FSac 5′ CTCAATGAGCTCCTCCCCAGGTCCCACAGCGCA.

HCNEx8-RSal 5′ CGGAGTGTCGACTGCTCTACAGAACAATACAAT.

The SacI/XhoI and SacI/SalI restriction sites were incorporated in the primers to facilitate cloning. PCR were carried out using mouse genomic DNA (C57Bl/6) as template. We used PfuTurbo Hotstart DNA polymerase (Agilent Technologies) following the protocol recommended by the manufacturer. The resulting fragments of 474 bp (exon 2) and 782 bp (exon 8) were cloned into the pmiRGlo using the restriction sites above. To generate mutant reporter constructs we conducted site directed mutagenesis using the QuickChange Site Directed Mutagenesis Kit (Agilent Technologies) following the manufacturer’s recommendation. Primers used for site directed mutagenesis were:

Exon 2 mutant F 5′-GTACTTCATCTTGATCCTCTGCGTTGCAAGGATGATTTCTGTGTTGTCC-3′

Exon 2 mutant R 5′-GGACAACACAGAAATCATCCTTGCAACGCAGAGGATCAAGATGAAGTAC-3′

Exon 8 mutant F 5′-CGCCAGGTTCCCTCTGGTTGGCAGCAGTAGTCAGAGG-3′

Exon 8 mutant R 5′-CCTCTGACTACTGCTGCCAACCAGAGGGAACCTGGCG-3′

The predicted exon 2 binding site was mutated from AUCAUCCUUG**ACC**CGCAGAGGA (wild-type) to AUCAUCCUUG**CAA**CGCAGAGGA (mutant) and the predicted exon 8 binding site was mutated from ACUACUGCUGC**ACC**CCAGAGGGA (wild-type) to ACUACUGCUGC**CAA**CCAGAGGGA—the corresponding nucleotides are in bold.

### Cell culture, transfection and luciferase reporter assays

We used H9C2 cardiac myoblast cell line for the luciferase reporter assay. Cells were maintained in Dulbecco's modified Eagle's medium (DMEM) (Invitrogen) supplemented with 10% foetal bovine serum and 1% penicillin/streptomycin. 5 × 10^5^ cells/well were plated in 24-well plates one day before the transfection. Cells were co-transfected with 0.5 µg of the luciferase reporter constructs and 0.5 μg of precursor miR-370-3p or negative control plasmid. Lipofectamine 2000 (Invitrogen) was used for all transfections according to the manufacturer’s instructions. Transfected cells were incubated with DNA:Lipofectamine complexes for 24 h before lysing in passive lysis buffer (Promega) for luciferase assay. Luciferase activity was determined using a Luciferase Assay System (Promega) following the manufacturer’s recommended protocol and detected using a luminometer (Berthold Technologies Lumat LB 9507). Luciferase assays were performed in quadruplicate and repeated three times with an independent batch of cells.

### Statistics

For qPCR of mouse cDNA involving microfluidic cards, outliers were automatically filtered using RealTime Statminer (v 4.1, Integromics). For qPCR of mouse cDNA involving 96-well plates, the median absolute deviation method was used to identify outliers. In all figures, means ± SEM are shown. Statistical differences were analysed using GraphPad Prism. Differences between two groups were analysed using Student’s unpaired t test for parametric data and the Mann–Whitney test for non-parametric data. Differences between more than two groups were analysed using one-way or two-way ANOVA followed by a suitable post hoc test in the case of parametric data and the Kruskal–Wallis test followed by a suitable post hoc test in the case of non-parametric data. Details of tests are given in figure legends.

## Supplementary information

Supplementary file1 (PDF 2349 kb)

## Data Availability

The datasets generated during and/or analysed during the current study are available from the corresponding author on reasonable request.
