# Supplementary material for: Silencing miR-370-3p rescues funny current and sinus node function in heart failure
Source: Sci Rep. 2020 Jul 9;10:11279. doi: 10.1038/s41598-020-67790-0 (PMC7347645; doi:10.1038/s41598-020-67790-0)
Supplement: Supplementary file 1 — Supplementary file1 (PDF 2349 kb) [file 41598_2020_67790_MOESM1_ESM.pdf]

SUPPLEMENTARY INFORMATION

Silencing miR-370-3p rescues funny current and sinus node function in heart failure

Joseph Yanni, Alicia D'Souza, Yanwen Wang, Ning Li, Brian J. Hansen, Stanislav O. Zakharkin, Matthew Smith, Christina Hayward, Bryan A. Whitson, Peter J. Mohler, Paul M.L. Janssen, Leo Zeef, Moinuddin Choudhury, Min Zi, Xue Cai, Sunil Jit R.J. Logantha, Shu Nakao, Andrew Atkinson, Maria Petkova, Ursula Doris, Jonathan Ariyaratnam, Elizabeth J. Cartwright, Sam Griffiths-Jones, George Hart, Vadim V. Fedorov, Delvac Oceandy, Halina Dobrzynski and Mark R. Boyett

Supplementary Discussion

HCN4 transcript expression normalised to the expression of a housekeeper transcript (as measured by qPCR) compared to I_f amplitude and I_f density. It is standard for electrophysiologists to normalise ionic current amplitude (I) to the cell capacitance, C_m, a measure of cell size (or more accurately cell surface area). This is for two reasons: first, because, the rate of change of membrane potential $\frac{dV}{dt} = {}^{I}/_{C_m}$. Secondly, for a given type of cell, it is known that ionic current amplitude depends on cell size, whereas ionic current density does not. This means that a larger cell will have more ion channels. In the absence of evidence to the contrary, it will be assumed that this is true of all transcripts and proteins, including housekeepers. In this case, ion channel transcript expression normalised to the expression of a housekeeper transcript should also be independent of cell size. In summary, what is driving membrane potential change is ionic current density and this is expected to reflect ion channel transcript expression normalised to housekeeper transcript expression as measured by qPCR (all other factors, such as post-translation modification etc. being constant). Based on this argument, in this study, HCN4 transcript expression (normalised to housekeeper transcript expression) was compared to I_f density rather than I_f amplitude.

2 mM Cs^+ as an I_f blocker. In this study, 2 mM Cs^+ was used as an I_f blocker. In rabbit sinus node cells, 2 mM Cs^+ completely blocks I_f in the pacemaker range of potentials[1,2]. This concentration of Cs^+ has a negligible effect on the L-type Ca^{2+} current, $I_{Ca,L}$, and the delayed rectifier K^+ current[1,2], two currents which play a major role in the sinus node action potential[3,4]. Cs^+ does block the background inward rectifier K^+ current, $I_{K,1}$. Isenberg[5] used 20 mM Cs^+ to block $I_{K,1}$ in sheep Purkinje fibres. It is unclear from the literature what fraction of $I_{K,1}$ will be blocked by 2 mM Cs^+. Harvey and Ten Eick[6] studied block of $I_{K,1}$ by Cs^+ in cat ventricular myocytes and reported that 1 mM Cs^+ blocked ~42% of the current at -100 mV. However, Cs^+ block of $I_{K,1}$ is voltage-dependent[6] and less block is expected in the pacemaker range of potentials. Furthermore, $I_{K,1}$ is not believed to play an important role in the centre of the sinus node (of the rabbit at least)[3,4]. We have previously discussed the use of 2 mM Cs^+ as an I_f blocker[7]. As an alternative to Cs^+, ivabradine could have been used to block I_f. However, ivabradine is a non-selective I_f blocker. 10 μM ivabradine decreases $I_{Ca,L}$ by 18% in rabbit sinus node cells[8]; see also Haechl *et al.*[9]. Ivabradine blocks heterologously expressed $Na_v1.5$ channels (responsible for the Na^+ current, I_{Na}, in the heart) with an IC_{50} of 30 μM[9] and Kv11.1 (hERG) channels (responsible for the important repolarizing current $I_{K,r}$ in the sinus node) with an IC_{50} of 2-3 μM[10] or 11 μM[9]. To minimise non-specific effects of ivabradine, it is advisable not to apply more than 3 μM ivabradine and yet at this concentration it only blocks ~50% of I_f; the IC_{50} for block of I_f is 2.8 μM[8]. It is concluded that, when using isolated sinus node tissue, 2 mM Cs^+ is the I_f blocker of choice, because it blocks all I_f and has minimal non-specific effects. However, neither I_f blocker is perfect.

Alternative forms of *miR-370-3p*. This study focusses on miR-370-3p, the commonest sequence of which is GCCUGCUGGGGUGGAACCUGGU. It is this form of miR-370-3p that we report from the TaqMan Array Rodent MicroRNA Card Set and individual qPCR assays (mouse and human); it is this form of miR-370-3p that we show to downregulate expression of HCN4 using the luciferase reporter gene assay; and it was this form that the antimiR used targets. This was the 439th most abundant microRNA in the dataset (abundance under control conditions, 0.049). It was this form of miR-370-3p that was significantly upregulated in the sinus node (by 50%). However, it is well known that there are often many different isoforms of mature microRNAs (isomiRs) present in the cell. RNAseq experiments capture this diversity (http://mirbase.org/cgi-bin/get_read.pl?acc=MI0001165). Of particular relevance here are isomiRs that may arise by the action of terminal uridylyl transferases (TUTases), which add uridyl residues to the 3' end of RNAs,

including miRNAs[11]. In the TaqMan Array Rodent MicroRNA Card Set, there is also an assay for a longer form of miR-370-3p with an extra uracil on the 3' end: GCCUGCUGGGGUGGAACCUGGUU. This was the 15th most abundant microRNA detected *(*abundance under control conditions, 0.235); the longer form was 4.8× more abundant than the short form. In response to heart failure, the longer form behaved in a similar manner to the shorter form: there was a trend (P=0.085) of an upregulation of the longer form (by 20%). Only one form of miR-139-3p was investigated.

Supplementary References

1 Liu, Y. M., Yu, H., Li, C.-Z., Cohen, I. S. & Vassalle, M. Cesium effects on i_f and i_K in rabbit sinoatrial node myocytes: implications for SA node automaticity. *Journal of Cardiovascular Pharmacology* **32**, 783-790 (1998).

2 Denyer, J. C. & Brown, H. F. Pacemaking in rabbit isolated sino-atrial node cells during Cs^+ block of the hyperpolarization-activated current i_f *Journal of Physiology* **429**, 401-409 (1990).

3 Brown, H. F. Electrophysiology of the sinoatrial node. *Physiological Reviews* **62**, 505-530 (1982).

4 Zhang, H., Holden, A. V., Kodama, I., Honjo, H., Lei, M., Varghese, T. & Boyett, M. R. Mathematical models of action potentials in the periphery and center of the rabbit sinoatrial node. *American Journal of Physiology* **279**, H397-H421 (2000).

5 Isenberg, G. Cardiac Purkinje fibers: cesium as a tool to block inward rectifying potassium currents. *Pflügers Archiv* **365**, 99-106 (1976).

6 Harvey, R. D. & Ten Eick, R. E. Voltage-dependent block of cardiac inward-rectifying potassium current by monovalent cations. *Journal of General Physiology* **94**, 349-361 (1989).

7 Nikmaram, M. R., Boyett, M. R., Kodama, I., Suzuki, R. & Honjo, H. Variation in the effects of Cs^+, UL-FS 49 and ZD7288 within the sinoatrial node. *American Journal of Physiology* **272**, H2782-H2792 (1997).

8 Bois, P., Bescond, J., Renaudon, B. & Lenfant, J. Mode of action of bradycardic agent, S 16257, on ionic currents of rabbit sinoatrial node cells. *British Journal of Pharmacology* **118**, 1051-1057 (1996).

9 Haechl, N., Ebner, J., Hilber, K., Todt, H. & Koenig, X. Pharmacological profile of the bradycardic agent ivabradine on human cardiac ion channels. *Cellular Physiology and Biochemistry* **53**, 36-48 (2019).

10 Melgari, D., Brack, K. E., Zhang, C., Zhang, Y., Harchi, A. E., Mitcheson, J. S., Dempsey, C. E., Ng, G. A. & Hancox, J. C. hERG potassium channel blockade by the HCN channel inhibitor bradycardic agent ivabradine. *Journal of the American Heart Association* **4**, e001813 (2015).

11 Thornton, J. E., Du, P., Jing, L., Sjekloca, L., Lin, S., Grossi, E., Sliz, P., Zon, L. I. & Gregory, R. I. Selective microRNA uridylation by Zcchc6 (TUT7) and Zcchc11 (TUT4). *Nucleic Acids Research* **42**, 11777-11791 (2014).

12 Miranda, K. C., Huynh, T., Tay, Y., Ang, Y. S., Tam, W. L., Thomson, A. M., Lim, B. & Rigoutsos, I. A pattern-based method for the identification of MicroRNA binding sites and their corresponding heteroduplexes. *Cell* **126**, 1203-1217 (2006).

13 Leslie, K. O., Taatjes, D. J., Schwarz, J., vonTurkovich, M. & Low, R. B. Cardiac myofibroblasts express alpha smooth muscle actin during right ventricular pressure overload in the rabbit. *American Journal of Pathology* **139**, 207-216 (1991).

14 Larkin, M. A., Blackshields, G., Brown, N. P., Chenna, R., McGettigan, P. A., McWilliam, H., Valentin, F., Wallace, I. M., Wilm, A., Lopez, R., Thompson, J. D., Gibson, T. J. & Higgins, D. G. Clustal W and Clustal X version 2.0. *Bioinformatics* **23**, 2947-2948 (2007).

15 Griffiths-Jones, S. RALEE--RNA ALignment editor in Emacs. *Bioinformatics* **21**, 257-259 (2005).

Supplementary Table 1. Echocardiographic measurements. LVIDd and LVIDs, left ventricular inner dimension during diastole and systole.

	**Control**	**Heart failure**	**Heart failure +antimiR**
**Left ventricular mass (mg)**	130.7 ± 5.0	253.9 ± 18.0	182.9 ± 12.4
**LVIDd (mm)**	4.4 ± 0.08	5.5 ± 0.2	4.9 ± 0.2
**LVIDs (mm)**	2.8 ± 0.08	4.8 ± 0.2	4.0 ± 0.3
**Fractional shortening (%)**	35.2 ± 1.2	13.2 ± 1.6	19.9 ± 3.2
**Ejection fraction (%)**	74.8 ± 1.3	33.4 ± 3.7	45.0 ± 8.1
**Stroke volume (μl)**	71.1 ± 3.5	55.5 ± 5.8	54.7 ± 7.6

Supplementary Table 2. Predicted binding sites within HCN4 mRNA for miR-139-3p, miR-145-5p, miR-351-3p and miR-370-3p. Cases of P<0.05 are highlighted in red. Analysed using RNA22[12].

Leftmost position of predicted target site	Folding energy (in kcal/mol) including contribution from linker	Predicted target site	Base pairing-mRNA	Base pairing-microRNA	P value
**mmu-miR-139-3p (TGGAGACGCGGCCCTGTTGGAG)**					
**512**	**-16.90**	**CGCCGCAGCCGGCCTCTGCCTCCT**	**(.((((((..((((..((.((((.**	**.)))).)).))))))).))).)**	**0.044200**
873	-13.90	CACCACACCCTGGATCGTCTTCA	..(((.((...((..((((((((	))))))))...))..))))))..	0.235000
2184	-15.20	CCTCAACTCAGGCGTCTTCA	...((((....(((((((((	)))))))))......))))...	0.094400
**2691**	**-22.70**	**CTCCACTGCCGCCGCCGCCTCCA**	**(((((.....(((((.(.(((((**	**))))).)))))).....)))))**	**0.000832**
2501	-22.60	CTTCAGCTCTGGGCTCTGCTTCA	(((((((...(((((.(((((((	))))).)).))))).)))))))	0.059000
**mmu-miR-145-5p (GTCCAGTTTTCCCAGGAATCCCT)**					
2910	-21.60	ATCCAGCCCTGGGCAGCTGGGC	.......((((((.((((((((	))))))))..))))))).......	0.067500
**mmu-miR-351-3p (GGTCAAGAGGCGCCTGGGAAC)**					
2750	-16.60	GCTCCCTGTCATCCTCTGACT	..((((.((...(((((((((	)))).))))).))..))))..	0.249000
2505	-15.60	AGCTCTGGGCTCTGCTTCACC	...((..(((.((.(((.(((	))).))).)).)))..))...	0.059000
**mmu-miR-370-3p (GCCTGCTGGGGTGGAACCTGGT)**					
33	-21.50	GCTCTACAGCCTTCCGCAGCAGGT	(((......((..((.((((((((	)))))))).)).)).....)))	0.285000
734	-16.90	AGAGGGTTAAGTCAGCAGGG	...(((((...((((((((.	.)))))))).....)))))...	0.201000
**976**	**-15.20**	**ATCATCCTTGACCCGCAGAGGA**	**((((...((.((((.((((((.**	**.))).))))))).))...))))**	**0.023100**
1310	-15.10	TCCTAGTGCCCATGCTGCAGGA	.....((..((((.(((((((.	.))))).)).)))).)).....	0.351000
1435	-21.10	ATTGGGTATGGACGGCAGGC	((..(((.(.(.((((((((	))))))))...).).)))..))	0.320000
1891	-13.30	AAGATGTACTTTATCCAGCACGGC	.....((.((...(((((((.((.	.)))))))))..)).)).....	0.133000
2527	-24.20	GCCAGCAGCCCCTCACAGGT	(((((...((((((((((((	))))).))))).))...)))))	0.059000
2570	-18.10	TCCACATCCAACAGCTGGC	.(((..((((.((((.(((	))).))))...))))...))).	0.059000
2952	-16.10	CCTAGGTCTGGCAGCTGGT	.(((((((((.((((.(((	))).))))...))).)))))).	0.183000
**3532**	**-20.30**	**ACTACTGCTGCACCCCAGAGGGA**	**((((..(.(.((((((((.(((.**	**.))).)))))))).).).))))**	**0.00483**

Supplementary Table 3. Patient information. AF, atrial fibrillation; CAD, coronary artery disease; ICH/CVA, intracranial haemorrhage/cerebral vascular accident; DM, diabetes mellitus; HTN, hypertension; ICD, implantable cardiac defibrillator; MI, myocardial infarction; PM, pacemaker; RA, right atrium; SAH, subarachnoid haemorrhage.

Heart number	Case number	Age	Gender	Heart weight (g)	Disease	Cause of death
**Control subjects**						
1	632941	68	Female	402	No cardiac disease	Natural causes CVA/Stroke
2	785258	51	Female	335	No cardiac disease	ICH/CVA
3	694855	46	Female	356	No cardiac disease	ICH/CVA and SAH
**Heart failure patients**						
4	514955	67	Female	566	Ischemic heart failure, CAD, HTN, DM, bradycardia, PM in RA	Transplant
5	369452	61	Male	540	Non-ischemic heart failure, CAD, HTN, ICD	Transplant
6	645444	47	Male	411	Ischemic heart failure, CAD, HTN, DM, ICD	Transplant
7	774694	50	Male	486	Ischemic heart failure, CAD, HTN, ICD	Transplant
8	674541	64	Male	599	Ischemic heart failure, HTN, DM, CAD MI, AF, ICD, PM in RA	Natural causes CVA/Stroke

Supplementary Table 4. *In vivo* administration of scrambled antimiR does not significantly impact body weight and cardiac structure or function. LVIDs, left ventricle internal diameter in systole; LVIDd, left ventricle internal diameter in diastole; LV mass, left ventricle mass. Data analysed using Student's t test.

	**Sham (n=6)**	**Sham + scrambled antimiR (n=5)**	***P* value**
Body weight (g)	27.3±0.7	28.4±0.9	0.71
Heart rate (conscious ECG) (beats/min)	752.8±11.5	757.8±9.5	0.14
LVIDs (mm)	2.60±0.14	2.88±0.12	0.18
LVIDd (mm)	4.34±0.03	4.43±0.05	0.22
LV mass (mg)	116.1±4.7	101.2±8.0	0.08
Fractional shortening (%)	40.0±2.3	35.0±2.1	0.14
Ejection fraction (%)	78.1±3.9	72.1±2.6	0.25
Stroke volume (µl)	66.4±3.0	65.7±1.8	0.83

Supplementary Table 5. Potential targets of mmu-miR-370-3p linked to cardiac hypertrophy. From Ingenuity Pathway Analysis.

Source	Confidence	Symbol	Name
TarBase, miRecords	Experimentally observed	MAP3K8	Mitogen-activated protein kinase kinase kinase 8
TargetScan Human	High (predicted)	MAP3K9	Mitogen-activated protein kinase kinase kinase 9
TargetScan Human	High (predicted)	GNAT1	Guanine nucleotide-binding protein G_t subunit alpha-1
TargetScan Human	Moderate (predicted)	GNG2	Guanine nucleotide-binding protein G_i/G_s/G_o subunit gamma-2
TargetScan Human	Moderate (predicted)	ADCY3	Adenylyl cyclase type 3
TargetScan Human	Moderate (predicted)	PRKACB	cAMP-dependent protein kinase catalytic subunit beta
TargetScan Human	Moderate (predicted)	CACNA1E	R-type Ca^{2+} channel
TargetScan Human	Moderate (predicted)	EIF2B1	Translation initiation factor eIF-2B subunit alpha

Supplementary Table 6. Sequences of individual microRNA real time primers used (all from Exiqon).

microRNA	Product number	Target sequence
mmu-miR-370-3p	204011	GCCUGCUGGGGUGGAACCUGGU
mmu-miR-139-3p	205162	UGGAGACGCGGCCCUGUUGGAG
U6 snRNA (mmu, hsa)	203907	GUGCUCGCUUCGGCAGCACAUAUACUAAAA UUGGAACGAUACAGAGAAGAUUAGCAUGG CCCCUGCGCAAGGAUGACACGCAAAUUCG UGAAGCGUUCCAUAUUUUU
RNU5G (mmu, hsa)	203908	AUACUCUGGUUUCUCUUCAGAUCGCAUAA AUCUUUCGCCUUUUACUAAAGAUUUCCG UGGAGAGGAACAACUCUGAGUCUUAACC CAAUUUUUUGAGCCUUGCUCCGACAAGGCUA

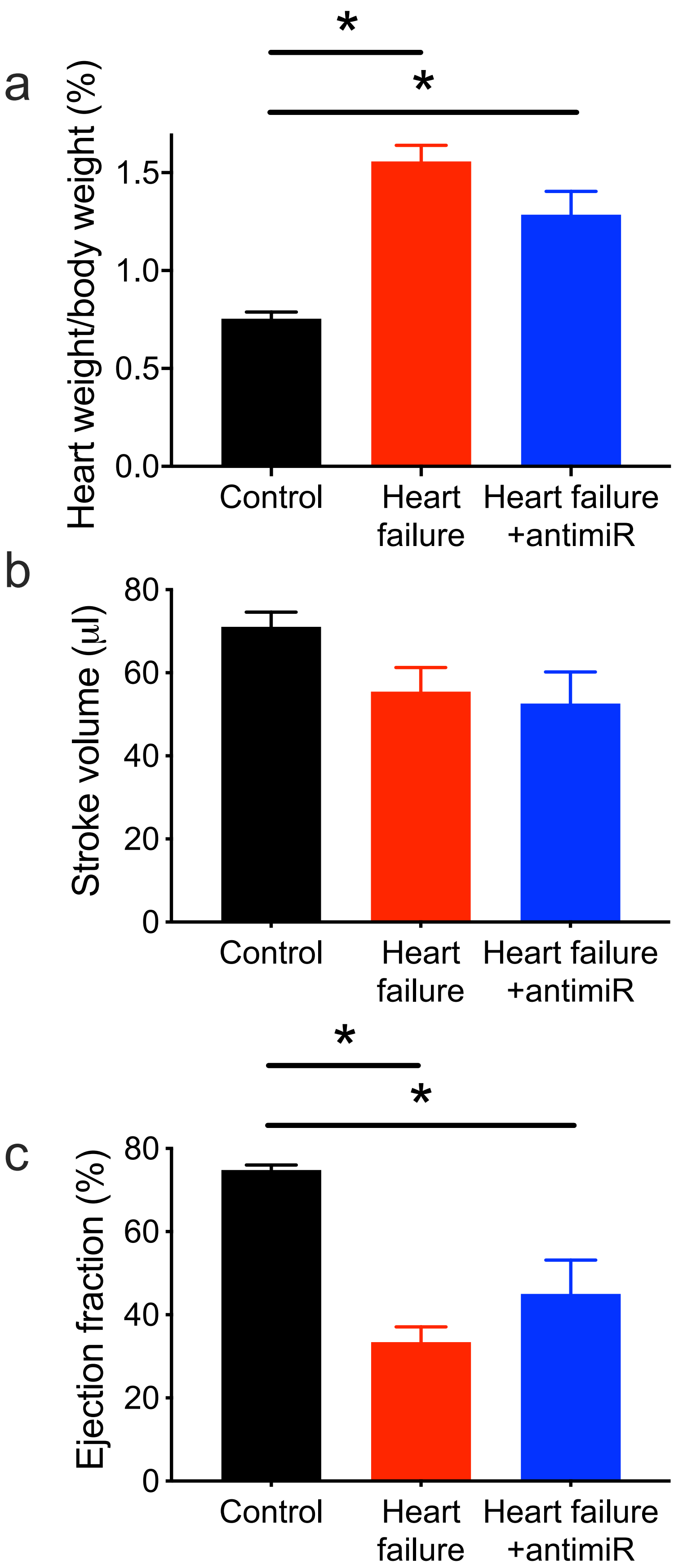

Supplementary Fig. 1. Heart weight to body weight ratio, stroke volume and ejection fraction of control mice given PBS, heart failure mice given PBS and heart failure mice given antimiR-370-3p. (**a**) Heart weight to body weight ratio (n=16, 19 and 10). (**b** and **c**) Stroke volume (n=17, 13 and 8) and ejection fraction (n=19, 13 and 10) calculated using echocardiography. *P<0.05; data analysed using one-way ANOVA and Tukey's multiple comparisons test.

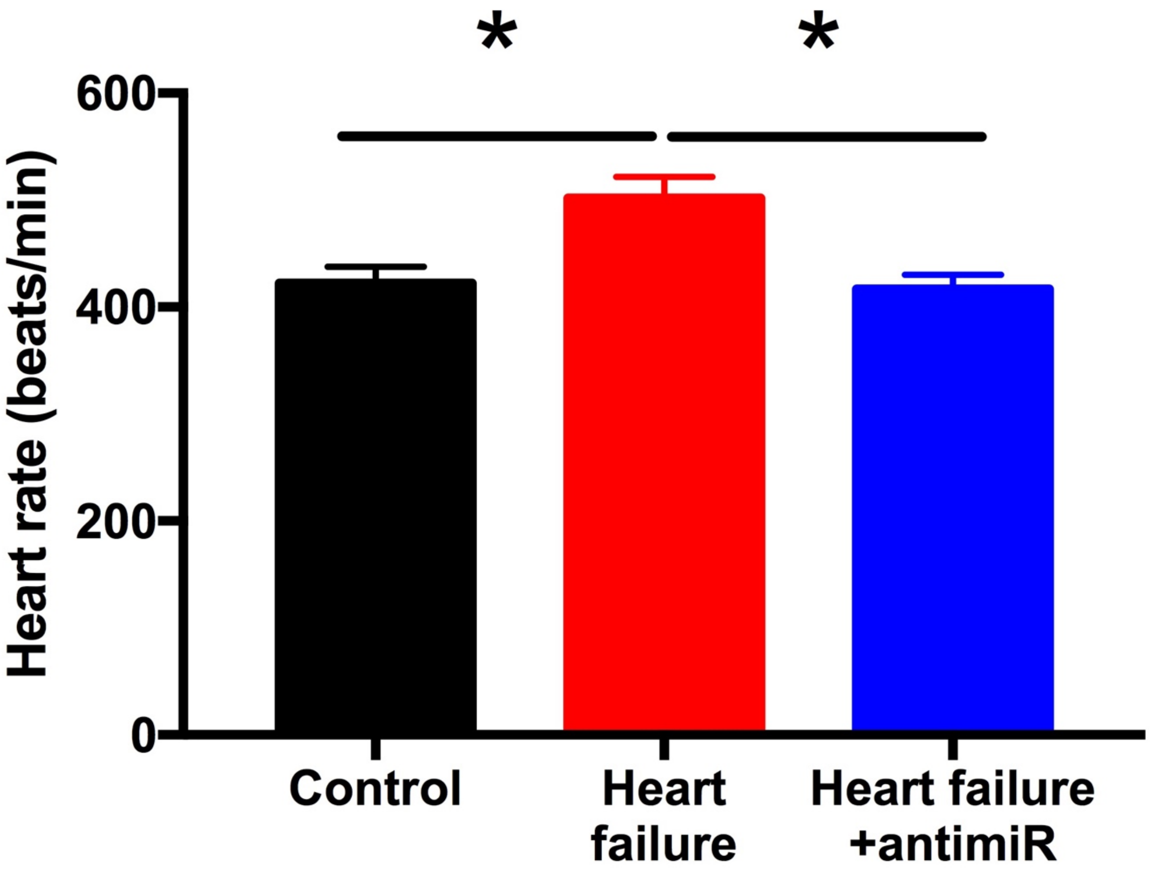

Supplementary Fig. 2. Heart rate (calculated from ECG of anaesthetised animals) of control mice given PBS, heart failure mice given PBS and heart failure mice given antimiR-370-3p (n=12, 14 and 10). *P<0.05; data analysed using one-way ANOVA and Tukey's multiple comparisons test.

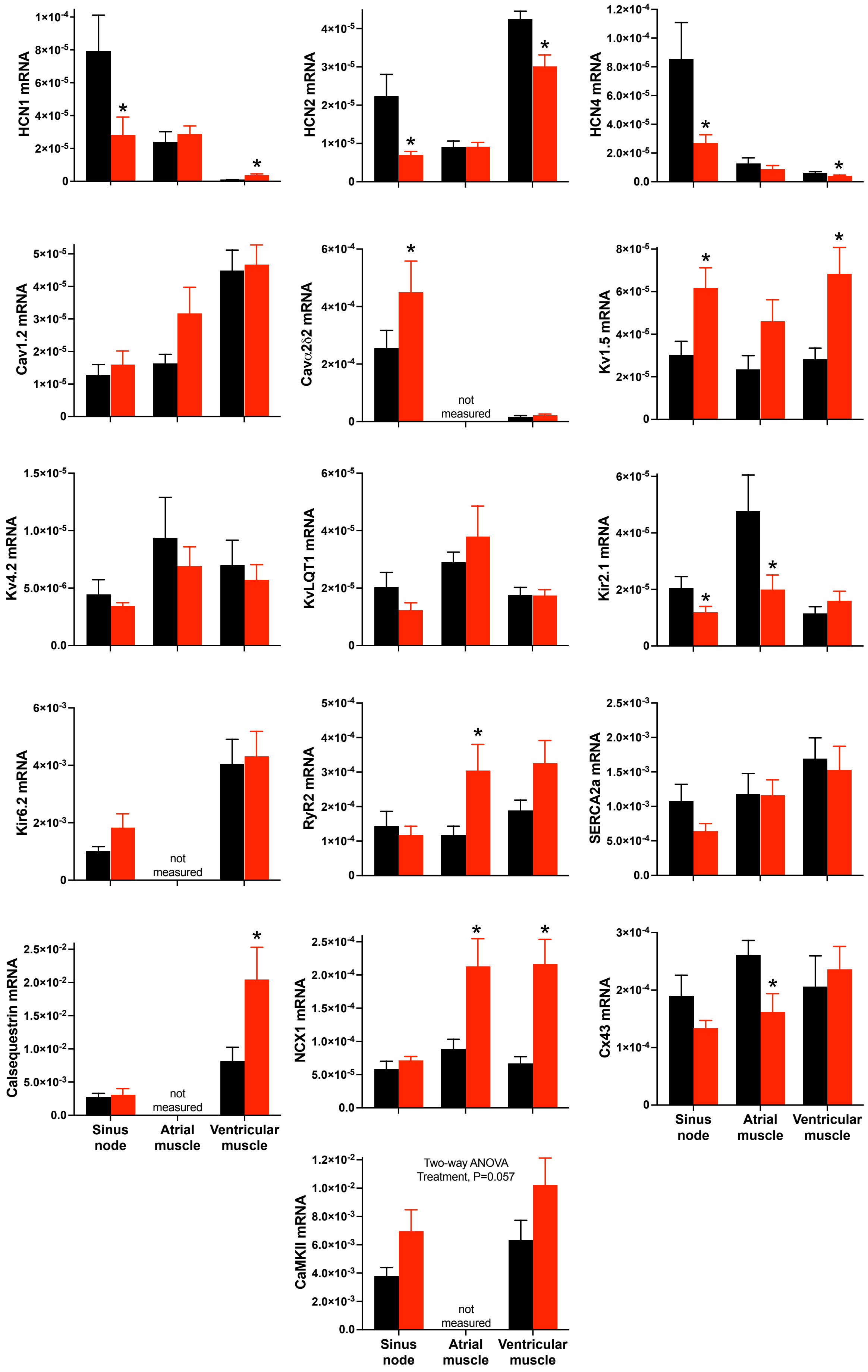

Supplementary Fig. 3. Expression of mRNA for selected ion channels and Ca^{2+}-handling molecules in the sinus node, atrial muscle and ventricular muscle in control and heart failure mice (n=7-10). Black bars, control mice; red bars, heart failure mice. *P<0.05 (heart failure versus control); data analysed using two-way ANOVA and Tukey's or Sidak's multiple comparisons test.

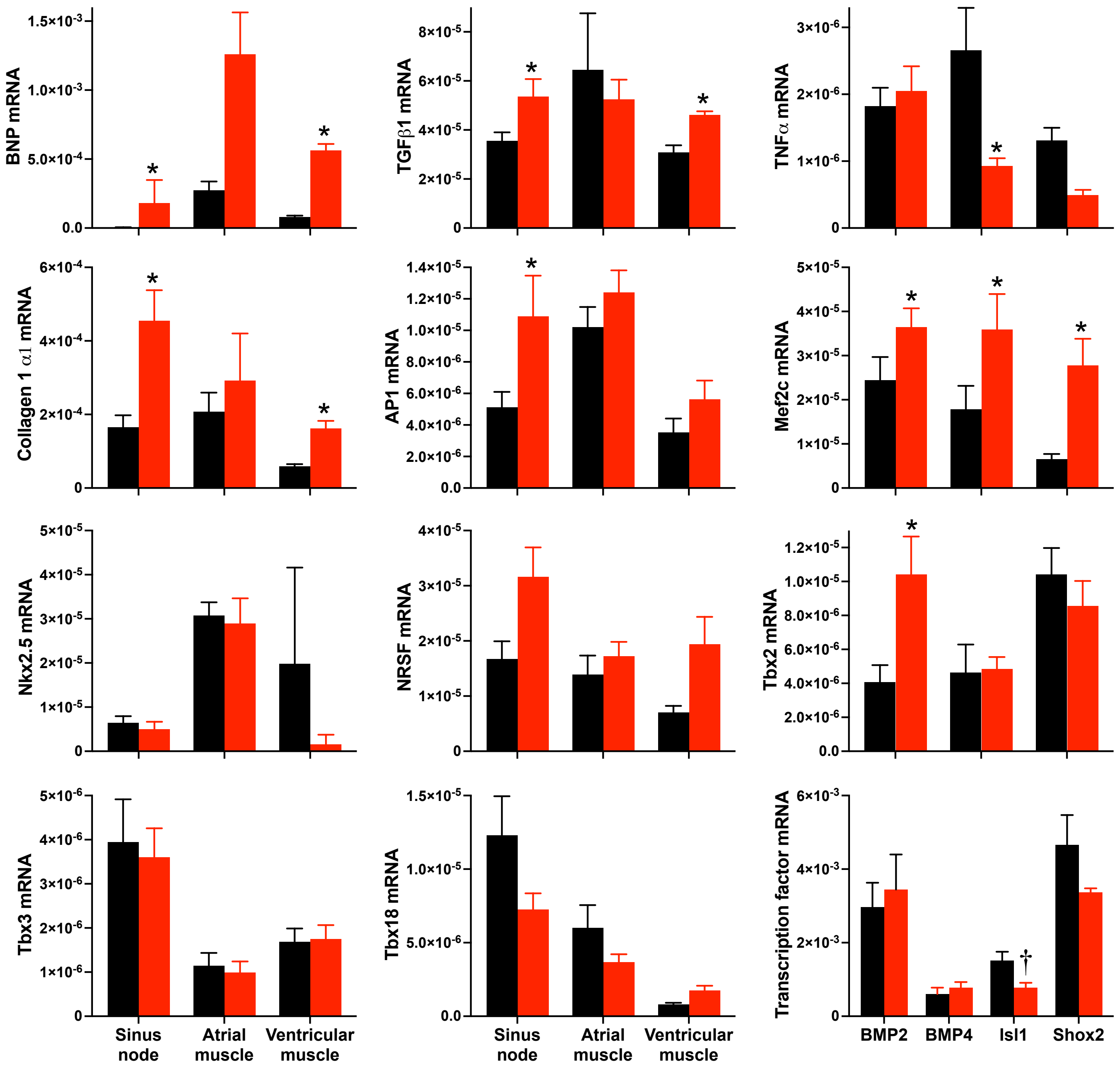

Supplementary Fig. 4. Expression of mRNA for a hypertrophy marker (BNP), selected fibrosis markers (TGFβ1, TNFα, collagen 1 α1) and transcription factors (AP1, Mef2c, Nkx2.5, NRSF, Tbx2, Tbx3, Tbx18, BMP2, BMP4, Isl1 and Shox2) in the sinus node, atrial muscle and ventricular muscle in control and heart failure mice. In all but the last panel, n=7-10. *P<0.05 (heart failure versus control); two-way ANOVA and Tukey's multiple comparisons test. In the last panel, transcripts measured in sinus node samples only (n=6/5, 5/5, 6/5 and 6/5 for BMP2, BMP4, Isl1 and Shox2, respectively). †P<0.05 (heart failure versus control); Student's unpaired t test. Black bars, control mice; red bars, heart failure mice.

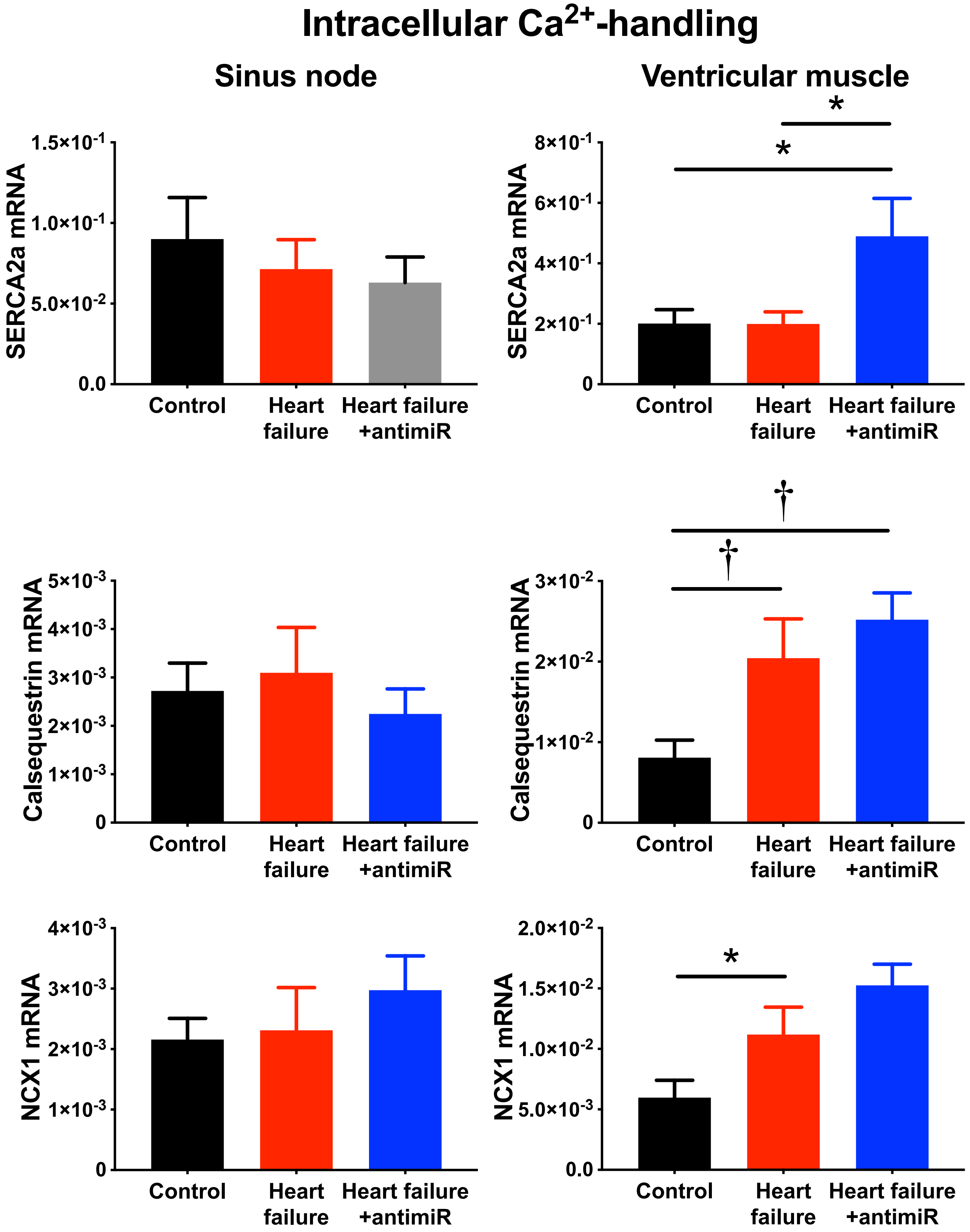

Supplementary Fig. 5. Expression of mRNA for molecules involved in intracellular Ca^{2+} handling (SERCA2, calsequestrin and NCX1) in the sinus node and left ventricle of control mice given PBS, heart failure mice given PBS and heart failure mice given antimiR-370-3p (n=9-13, 11-18 and 6-9). Control and heart failure data same as shown in Supplementary Fig. 3. *P<0.05; data analysed using one-way ANOVA and Tukey's multiple comparisons test. In the case of calsequestrin expression in the left ventricle, one-way ANOVA showed that there are significant differences among the means, but the post hoc test did not reveal these differences; however, Student's unpaired t-test showed significant differences (†P<0.05).

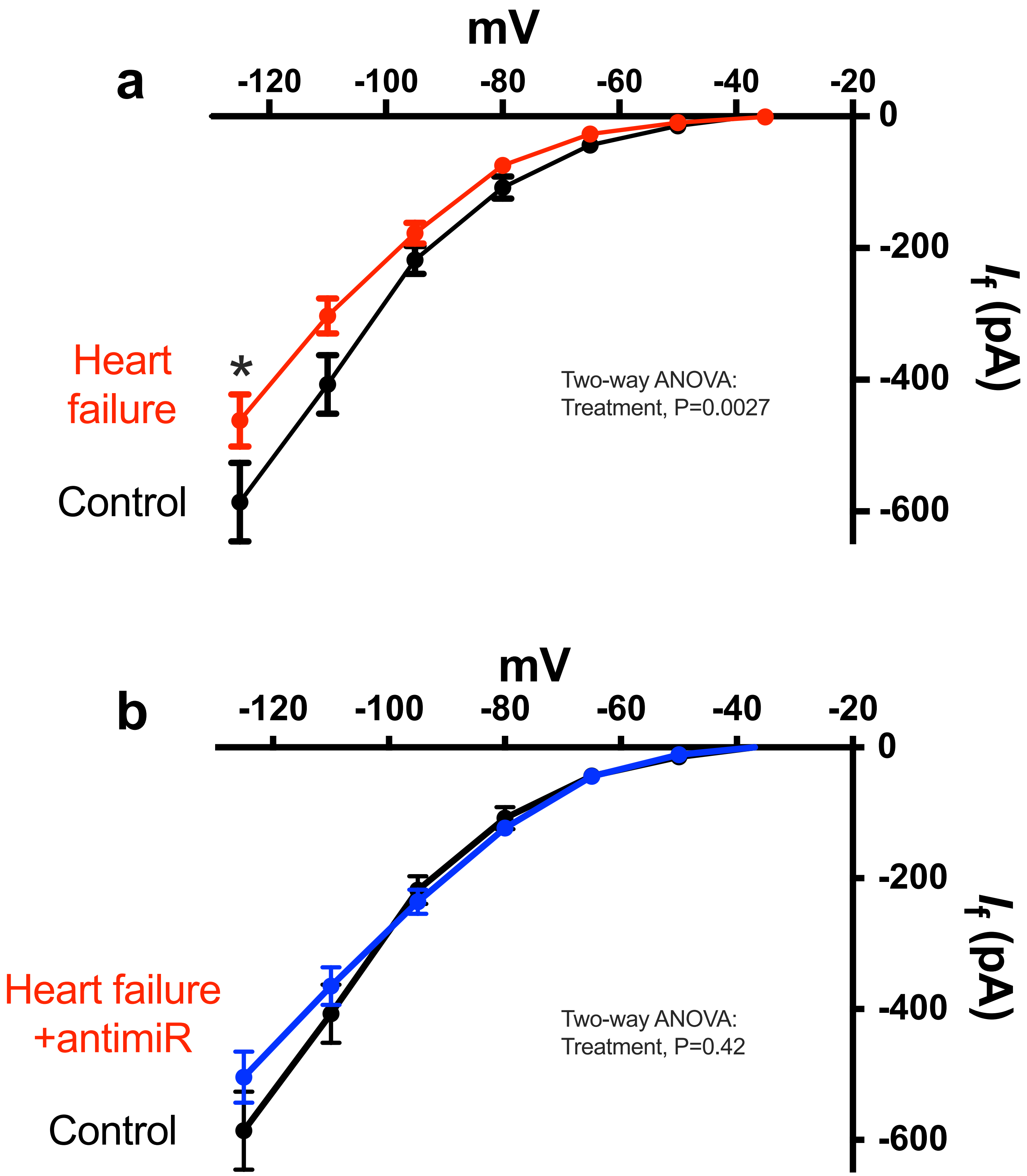

Supplementary Fig. 6. Mean current-voltage relationships for I_f (current amplitude in pA shown) from sinus node cells. (**a**) Comparison of control mice given PBS with heart failure mice given PBS (n=11 and 40 cells from 3 and 4 mice). I_f amplitude in heart failure mice significantly less than in control mice (P= 0.0027; two-way ANOVA). *P<0.05 (Sidak's multiple comparisons test). (**b**) I_f amplitude in control mice and heart failure mice given antimiR-370-3p (n=11 and 46 cells from 3 and 5 mice). I_f amplitude in the two groups of mice not significantly different (P=0.42; two-way ANOVA).

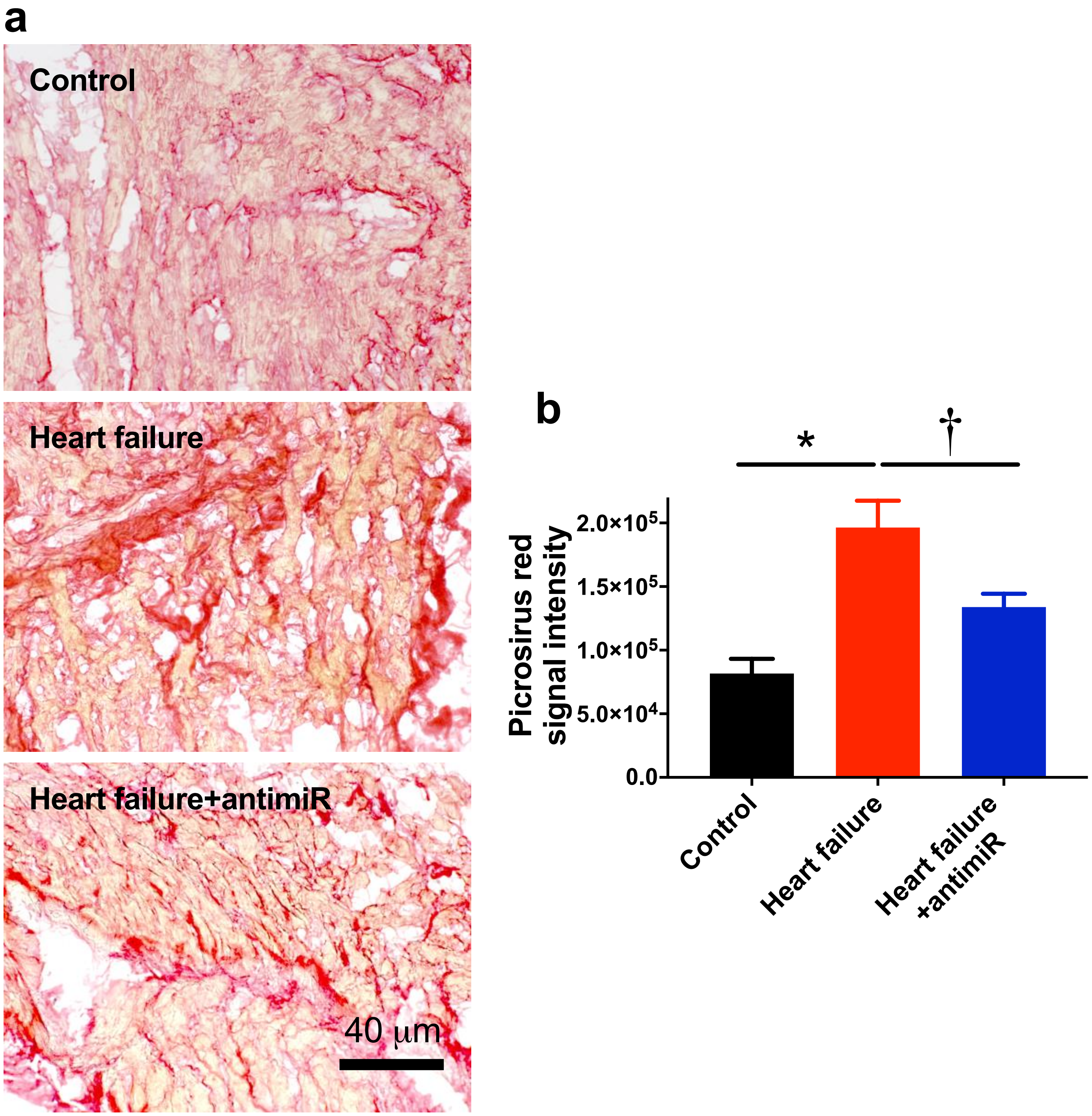

Supplementary Fig. 7. Silencing miR-370-3p improves fibrosis of atrial muscle in heart failure. (**a**) Images of picrosirius red stained tissue sections through atrial muscle of a control mouse given PBS, a heart failure mouse given PBS and a heart failure mouse given antimiR-370-3p. Red stain, collagen; yellow stain, cardiac myocytes. (**b**) Mean intensity of picrosirius red staining of collagen (red signal) in the three groups of mice (n=30, 29 and 48 sections from 4, 3 and 3 mice). *$P<0.05$; Kruskal-Wallis test followed by Dunn's multiple comparisons test. †$P<0.05$; data analysed using Student's unpaired t-test.

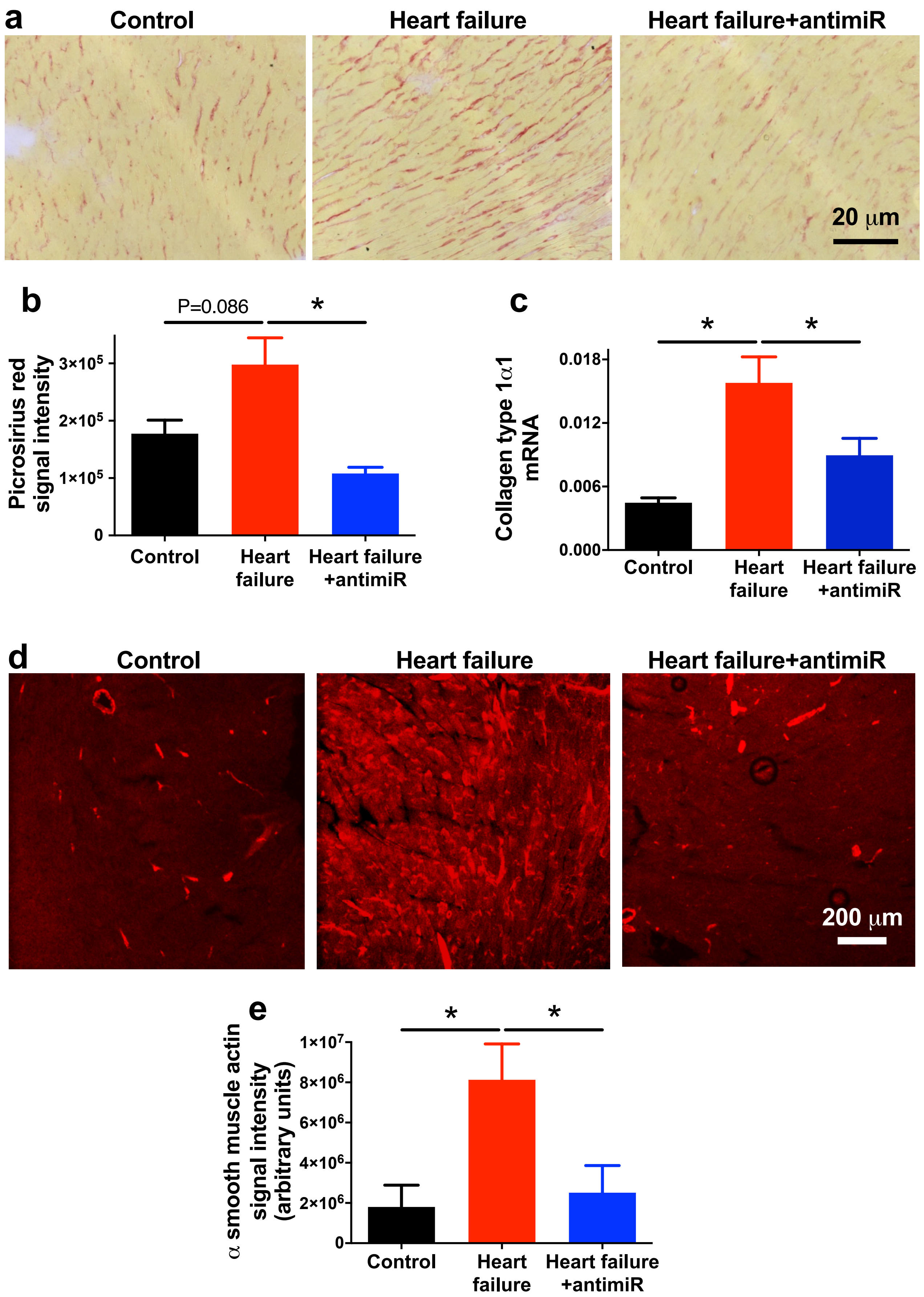

Supplementary Fig. 8. Silencing miR-370-3p improves fibrosis of ventricular muscle in heart failure. (**a**) Images of picrosirius red stained tissue sections through ventricular muscle of a control mouse given PBS, a heart failure mouse given PBS and a heart failure mouse given antimiR-370-3p. Red stain, collagen; yellow stain, cardiac myocytes. (**b**) Mean intensity of picrosirius red staining of collagen (red signal) in the three groups of mice (n=4, 6 and 4). *P<0.05; data analysed using

one-way ANOVA and Holm-Sidak multiple comparisons test. The difference between the control and heart failure groups was analysed using Student's unpaired t-test; the P value is given. (**c**) Expression of collagen type 1 α1 mRNA in the three groups of mice (n=8, 9 and 7). *P<0.05; data analysed using one-way ANOVA and Tukey's multiple comparisons test. (**d**) Images of tissue sections through ventricular muscle of a control mouse given PBS, a heart failure mouse given PBS and a heart failure mouse given antimiR-370-3p immunolabelled for α smooth muscle actin (red signal). In right ventricular pressure overload in the rabbit, α smooth muscle actin is expressed by myofibroblasts, which are likely to be responsible for the increase in interstitial collagen[13]. (**e**) Mean intensity of α smooth muscle actin immunolabelling in the three groups of mice (n=3, 3 and 4). *P<0.05; data analysed using one-way ANOVA and Tukey's multiple comparisons test.

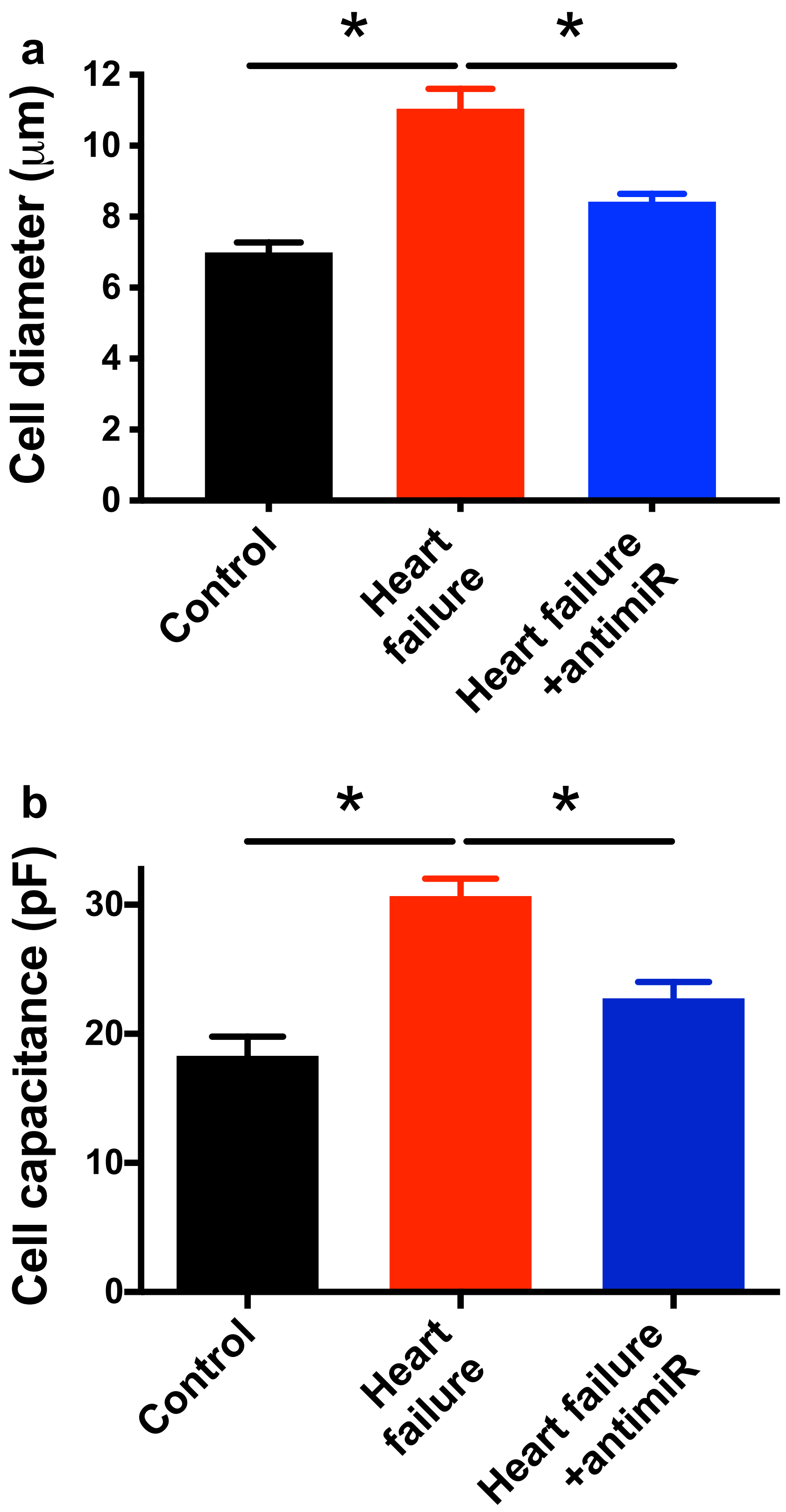

Supplementary Fig. 9. Silencing miR-370-3p improves hypertrophy of sinus node cells in heart failure. (**a**) Diameter of sinus node cells isolated from control mice given PBS (n=20 cells; 5 mice), heart failure mice given PBS (n=32 cells; 5 mice) and heart failure mice given antimiR-370-3p (n=20 cells; 5 mice). *P<0.05; one-way ANOVA followed by Dunn's multiple comparisons test. (**b**) Capacitance of sinus node cells isolated from control mice given PBS (n=11 cells; 3 mice), heart failure mice given PBS (n=44 cells; 5 mice) and heart failure mice given antimiR-370-3p (n=51 cells; 5 mice). *P<0.05; Kruskal-Wallis test followed by Dunn's multiple comparisons test. Cell diameter and capacitance was measured using the same batches of cells (either spindle- or elongated spindle-shaped cells used). Cell diameter was measured from HCN4 immunolabelled cells and cell capacitance measured during patch clamp experiments.

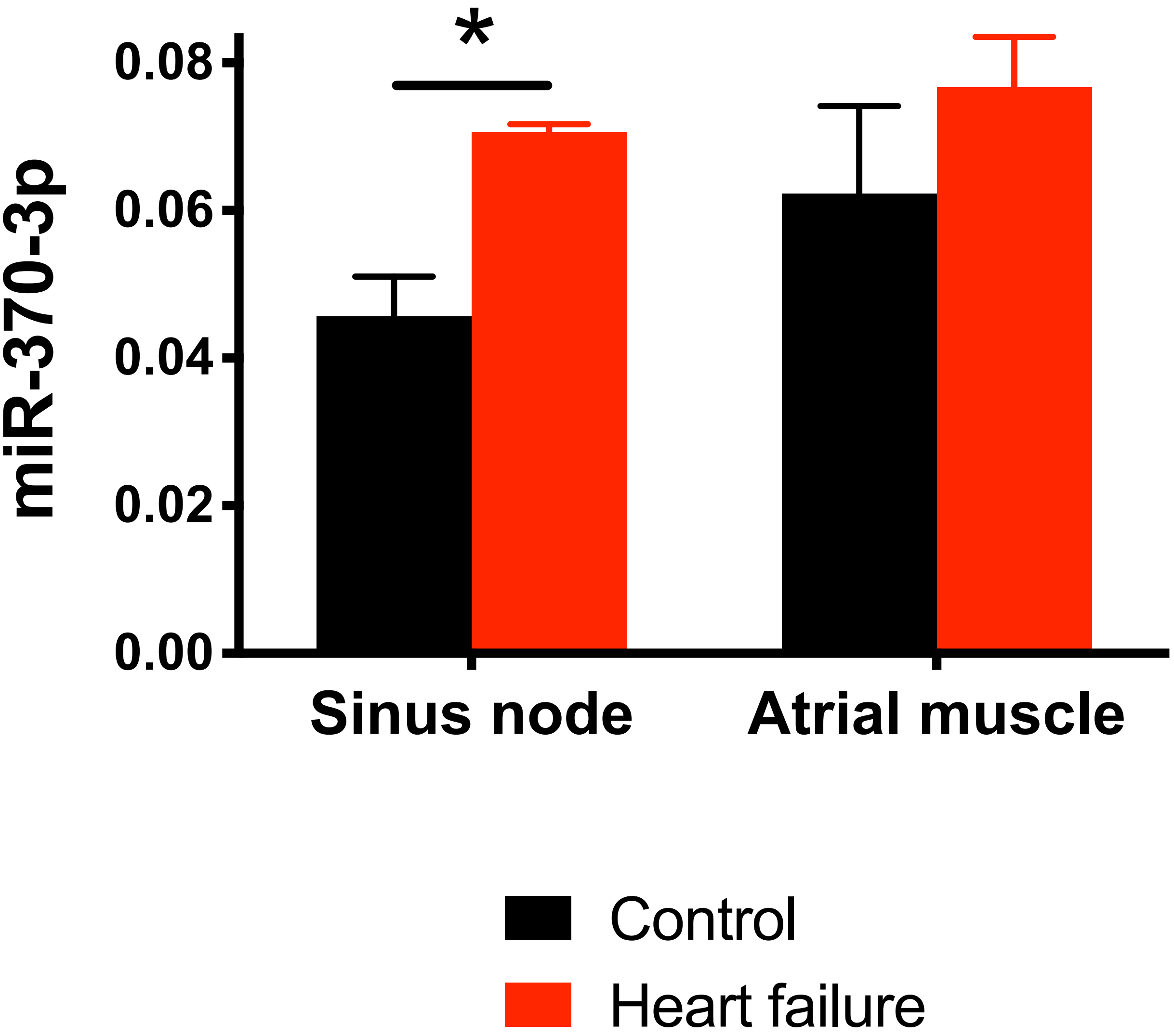

Supplementary Fig. 10. Heart failure causes significant upregulation of miR-370-3p in the sinus node, but not atrial muscle. Expression of miR-370-3p shown in sinus node and atrial muscle in control and heart failure mice (n=8 and 10). *P<0.05; one-way ANOVA followed by Holm-Sidak multiple comparisons test.

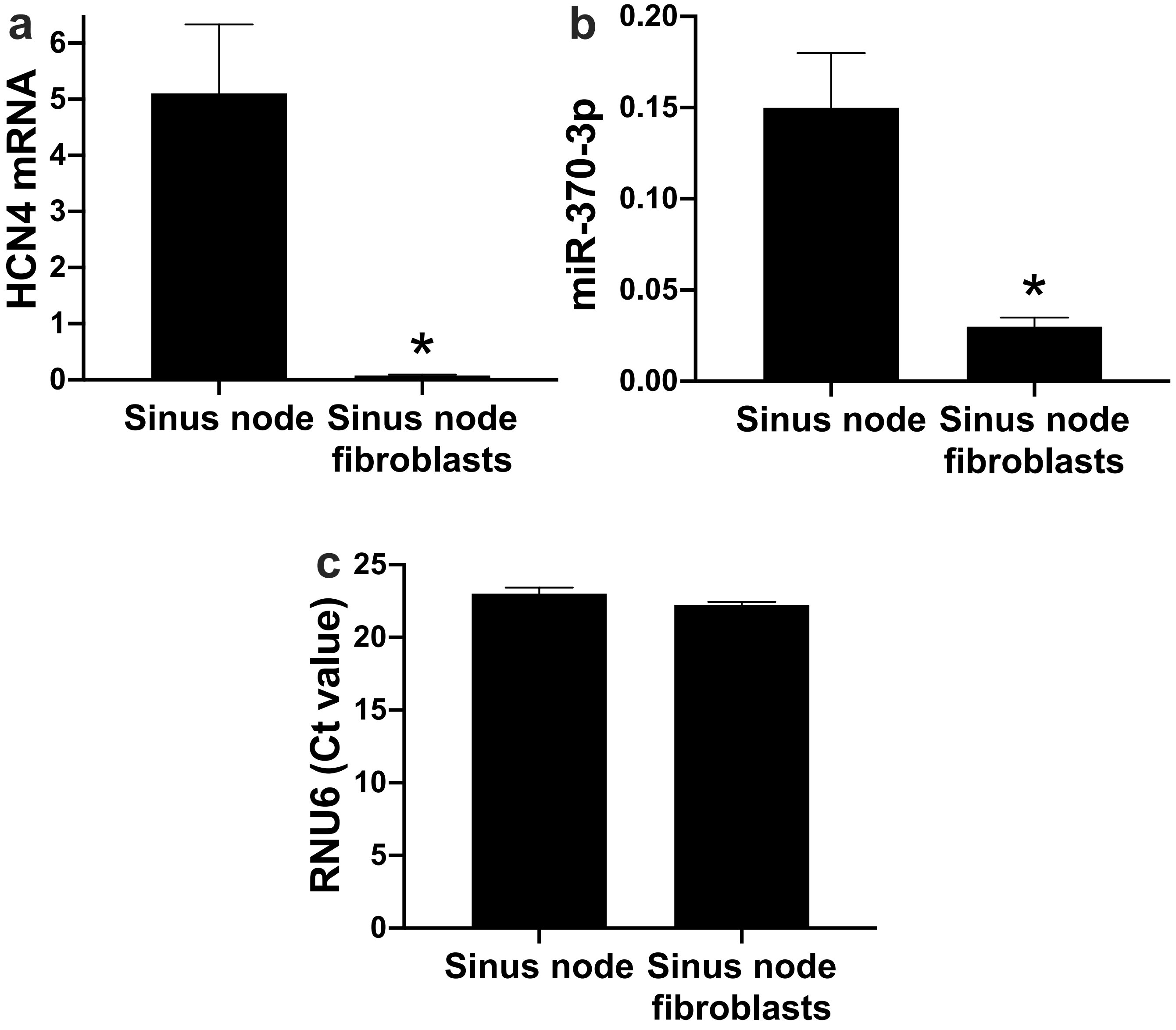

Supplementary Fig. 11. (**a**, **b**) mRNA expression of HCN4 (a) and miR-370-3p (b) in the intact sinus node and fibroblasts isolated from the sinus node. (**c**) Ct values for reference gene RNU6 in the intact sinus node and fibroblasts isolated from the sinus node. Intact sinus node preparations: n=3. Fibroblast preparations: n=2 independent preparations each from 3 pooled sinus node biopsies. *P<0.05; Student’s unpaired t-test.

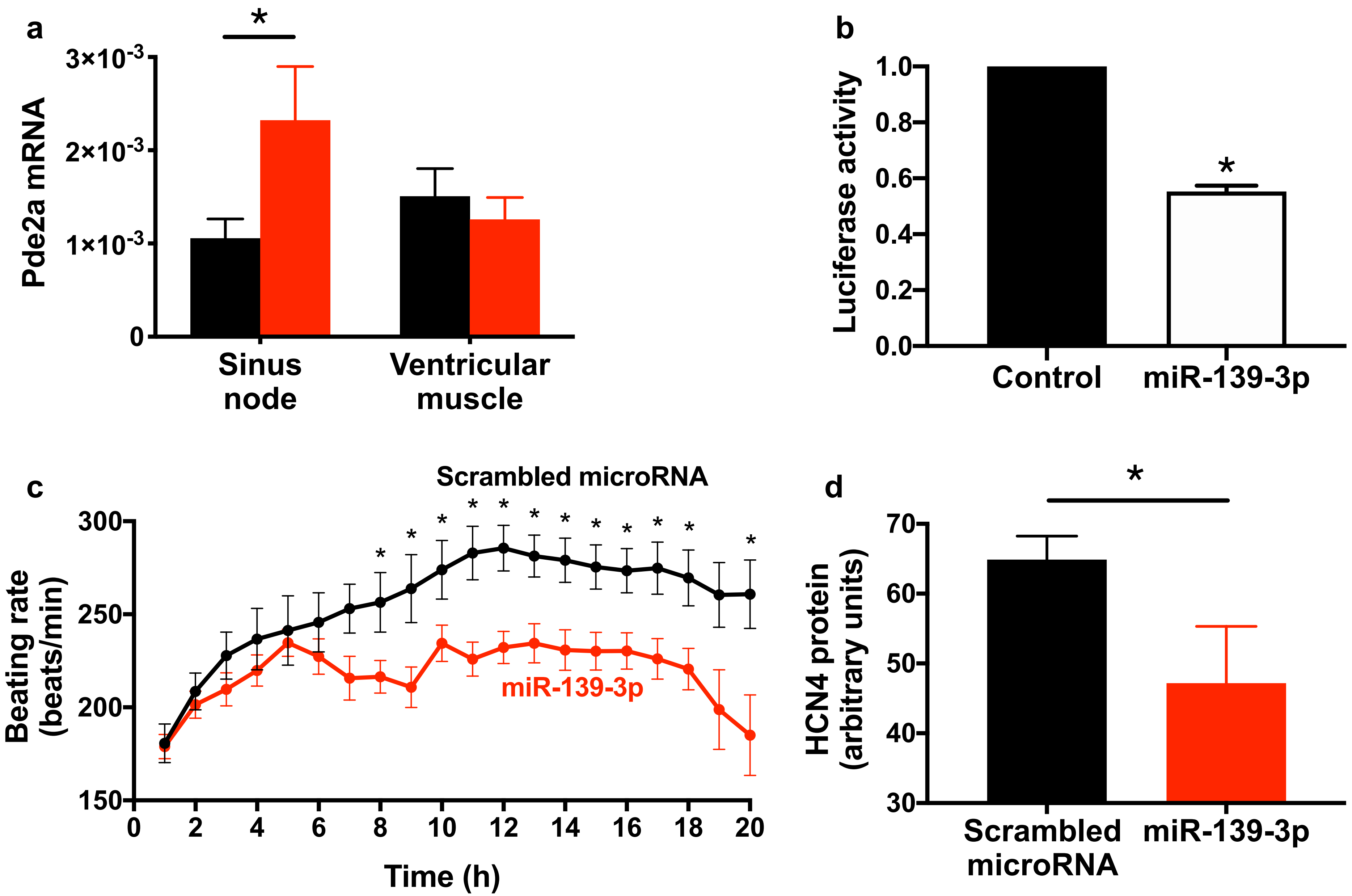

Supplementary Fig. 12. Source and function of miR-139-3p. (**a**) Expression of Pde2a mRNA in the sinus node and left ventricle of control mice given PBS and heart failure mice given PBS (n=9 or 14 and 10 or 15). *P<0.05; two-way ANOVA followed by Sidak multiple comparisons test. (**b**)

Evidence of binding of miR-139 to the 3' untranslated region (UTR) of HCN4 mRNA using the luciferase reporter gene assay. The 3'-UTR of HCN4 was cloned downstream of the luc2 firefly luciferase gene under the control of the PGK (phosphoglycerate kinase 1) promoter. H9C2 cells were co-transfected with this construct and a plasmid containing the microRNA of interest. The graph shows a ratio of luciferase activity (bioluminescence) normalised to Renilla bioluminescence. The control involved a plasmid containing a scrambled microRNA sequence that does not bind specifically to the HCN4 3′-UTR. n=3 batches of cells with 4 replicates/batch. *P<0.05; one-way ANOVA. (**c**) Time course of the beating rate of isolated sinus node preparations following the injection of a scrambled microRNA or miR-139-3p (n=7 and 8). *P<0.05; data analysed using two-way ANOVA followed by the Holm-Sidak multiple comparisons test. (**d**) Mean intensity of immunolabelling of HCN4 in the sinus node of isolated sinus node preparations that had been injected with a scrambled microRNA or miR-139-3p 24 h previously (n=4 and 4 rats; 8 sections/rat). *P<0.05; Student's unpaired t-test.

```
                        5p                                   3p
mmu-mir-370      agacg.gagagacCAGGUCACGUCUCUGCAGUUacacagcucaugaguGCCUGCUGGGGUGGAACCUGGUuugucu
rno-mir-370      agaca.gagagacCAGGUCACGUCUCUGCAGUUACACagcucaugaguGCCUGCUGGGGUGGAACCUGGUuugucu
hsa-mir-370      agaca.gagaagcCAGGUCACGUCUCUGCAGUUACacagcucacgaguGCCUGCUGGGGUGGAACCUGGUcugucu
ppy-mir-370      agaca.gagaagccaggucacgucucugcaguuacacagcucacgaguGCCUGCUGGGGUGGAACCUGGUcugucu
ptr-mir-370      .gaca.gagaagccaggucacgucucugcaguuacacagcucacgaguGCCUGCUGGGGUGGAACCUGGUcugucu
bta-mir-370      aggca.gagaagccaggucacgucucugcaguuacaccgcucacgaguGCCUGCUGGGGUGGAACCUGGUcugucu
oar-mir-370      aggca.gagaagcCAGGUCACGUCUCUGCAGUUACaccgcucucgaguGCCUGCUGGGGUGGAACCUGGUCUgucu
ssc-mir-370      agaca.gagaagccaggucacgucucugcaguuacacagcucacgaguGCCUGCUGGGGUGGAACCUGGUcugucu
mml-mir-370      agaca.gagaagcCAGGUCACGUCUCUGCAGUUACacagcucaugaguGCCUGCUGGGGUGGAACCUGGUcugucu
eca-mir-370      agaca.gagaggccaggucacgucucugcaguuacacagcucaugaguGCCUGCUGGGGUGGAACCUGGUcugucu
cfa-mir-370      agaca.gagaggccaggucacgucucugcaguuacacagcucaugaguGCCUGCUGGGGUGGAACCUGGUcugucu
efu-mir-370      gcacaagacaggccaggucacgucucugcaguuacacagcucaugaguGCCUGCUGGGGUGGAACCUGGUcugucu
#=GC SS_cons     (((((......(((((((..(..(((.((((.(((.(.......).))).)))).)))..)..))))))).)))))
```

Supplementary Fig. 13. Sequence of miR-370-3p is conserved in different species. A multiple sequence alignment of the miR-370 precursor is shown with sequences from the mouse (mmu), rat (rno), human (hsa), orangutan (ppy), chimpanzee (ptr), cow (bta), sheep (oar), wild boar (ssc), rhesus monkey (mml), horse (eca), dog (cfa) and bat (efu). Mature sequences of miR-370-5p and miR-370-3p are in uppercase. The SS_cons line represents the consensus secondary structure of the miRNA hairpin in dot-bracket notation. The alignments are from ClustalW[14], visualised in RALEE[15].

```
                              5p                                        3p
mmu-mir-139      guguauUCUACAGUGCACGUGUCUCCAGuguggcucgg...aggcUGGAGACGCGGCCCUGUUGGA.Guaa
rno-mir-139      guguauUCUACAGUGCACGUGUCUCCAGuguggcucgg...aggcUGGAGACGCGGCCCUGUUGGA.Guaa
cgr-mir-139      guguauUCUACAGUGCACGUGUCUCCAGuguggcucag...aggcUGGAGACGCGGCCCUGUUGGA.GUaa
hsa-mir-139      guguauUCUACAGUGCACGUGUCUCCAGUguggcucgg...aggcUGGAGACGCGGCCCUGUUGGA.GUaa
ggo-mir-139      guguauUCUACAGUGCACGUGUCUCCAGuguggcucgg...aggcuggagacgcggcccuguugga.guaa
ppa-mir-139      guguauUCUACAGUGCACGUGUCUccaguguggcucgg...aggcuggagacgcggcccuguugga.guaa
ptr-mir-139      guguauucuacagugcacgugucuccaguguggcucgg...aggcuGGAGACGCGGCCCUGUUGGA.GUaa
efu-mir-139      guguauucuacagugcacgugucuccaguguggcucgg...aggcUGGAGACGCGGCCCUGUUGGA.GUaa
lla-mir-139      guguauUCUACAGUGCACGUGUCUccaguguggcucgg...aggcuggagacgcggcccuguugga.guaa
ppy-mir-139      guguauUCUACAGUGCACGUGUCUCCAGuguggcucgg...aggcuGGAGACGCGGCCCUGUUGGA.GUaa
mml-mir-139      guguauUCUACAGUGCACGUGUCUCCAGuguggcucgg...aggcuGGAGACGCGGCCCUGUUGGA.GUaa
ssc-mir-139      guguauUCUACAGUGCACGUGUCUCCAGuguggcucgg...aggcUGGAGACGCGGCCCUGUUGGA.GUaa
bta-mir-139      guguacUCUACAGUGCACGUGUCUCCAGUguggcucgg...aggcuggagacgcggcccuguugga.guaa
tch-mir-139      ......UCUACAGUGCACGUGUCUCCAGUguggcucag...aggcuggagacgcggcccugucgga.gu..
cfa-mir-139      .......cuacagugcacgugucuccaguguggcuccg...aggcUGGAGACGCGGCCCUGUUGGA.A...
eca-mir-139      ...uacUCUACAGUGCACGUGUCUCCAGuguggcuccg...aggcuGGAGACGCGGCCCUGUUGGA.GUa.
mdo-mir-139      .uguauUCUACAGUGCAUGUGUCUccagugugacuaag...ggacUGGAGAUACAGCCCUGUUGGA.AUaa
sha-mir-139      guguauUCUACAGUGCAUGUGUCUCCAGUgugacuaag...ggacuggagauacggcccuguugga.auaa
tgu-mir-139      cuguauUCUACAGUGCAUGUGUCUCCAGUgugaguaag...ugacUGGAGAUGCGGCCCUGUUGGA.AUaa
ola-mir-139      cuguauUCUACAGUGCAUGUGUCUCCAguauguuagugaugcuacuggagacucggcucuguugga.auaa
ssa-mir-139-1    ......UCUACAGUGCAUGUGUCUCCAGU..guuugug.ugcuacUGGAGAUAUGGCUCUGUUGGA.AU..
aca-mir-139      cuguauUCUACAGUGCAUGUGUCUCCAGU..Guuuucc.agcgacUGGAGAUACGGCCCUGUUGGA.auaa
oha-mir-139      uuguauUCUACAGUGCAUGUGUCUCCAGCGuguuuaag...cagcUGGAGAUACGGCCCUGUUGGA.auaa
xtr-mir-139      .uguauUCUACAGUGCAUGUGUCUccagucauauagag...gcacuggggauacagcucuguugga.auaa
oan-mir-139      guguagUCUACAGUGCAUGUGUCUCCAGggacacuaga...cagcUGGAGACACAGCUCUGUUGGAUacaa
ccr-mir-139      cuguauUCUACAGUGCAUGUGUCUCCAGUguuucuaug..gcgacuggggaggcagcgcuguugga.auaa
ipu-mir-139      ......UCUACAGUGCAUGUGUCUCCAGUguuucuaug..gcgacuggggaggcagcgcuguugga.au..
dre-mir-139      cuguauUCUACAGUGCAUGUGUCUccaguguuucuaug..gcgacUGGGGAGGCAGCGCUGUUGGA.AUaa
ssa-mir-139-2    ......UCUACAGUGCAUGUGUCUCCAGUgaauaug.a..gcuacUGGAGAGGCAGUUCUGUGUGA.GU..
#=GC SS_cons     .(((..(((((((.((.((((((((((((..............)))))))))))))).)))).))).))).
```

Supplementary Fig. 14. Sequence of miR-139-3p is conserved. A multiple sequence alignment of the miR-139 precursor is shown with sequences from the mouse (mmu), rat (rno), Chinese hamster (cgr), human (hsa), gorilla (ggo), bonobo formerly called the pygmy chimpanzee (ppa), common chimpanzee (ptr), bat (efu), brown woolly monkey (lla), orangutan (ppy), rhesus monkey (mml), wild boar (ssc), cow (bta), Chinese tree shrew (tch), dog (cfa), horse (eca), gray short-tailed opossum (mdo), Tasmanian devil (sha), Chinese tree shrew (tgu), Japanese rice fish (ola), Atlantic salmon (ssa), lizard Carolina anole (aca), king cobra (oha), xenopus (xtr), platypus (oan), common carp (ccr), channel catfish (ipu) and zebrafish (dre). Mature sequences of miR-139-5p and miR-139-3p are in uppercase. The SS_cons line represents the consensus secondary structure of the miRNA hairpin in dot-bracket notation. The alignments are from ClustalW[14], visualised in RALEE[15].

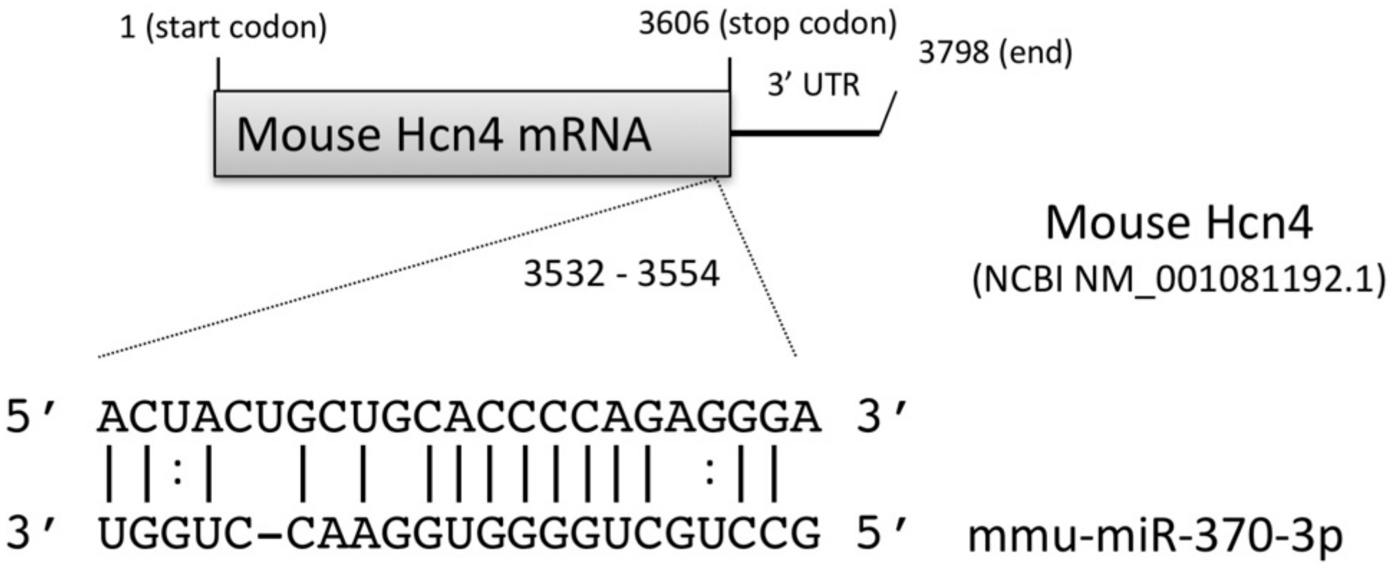

Supplementary Fig. 15. Predicted miR-370-3p binding site in exon 8 of HCN4. In the mutant, the predicted exon 8 binding site was mutated from ACUACUGCUGC**ACC**CCAGAGGGA (wild-type) to ACUACUGCUGC**CAA**CCAGAGGGA (mutant) – the corresponding nucleotides are in bold.

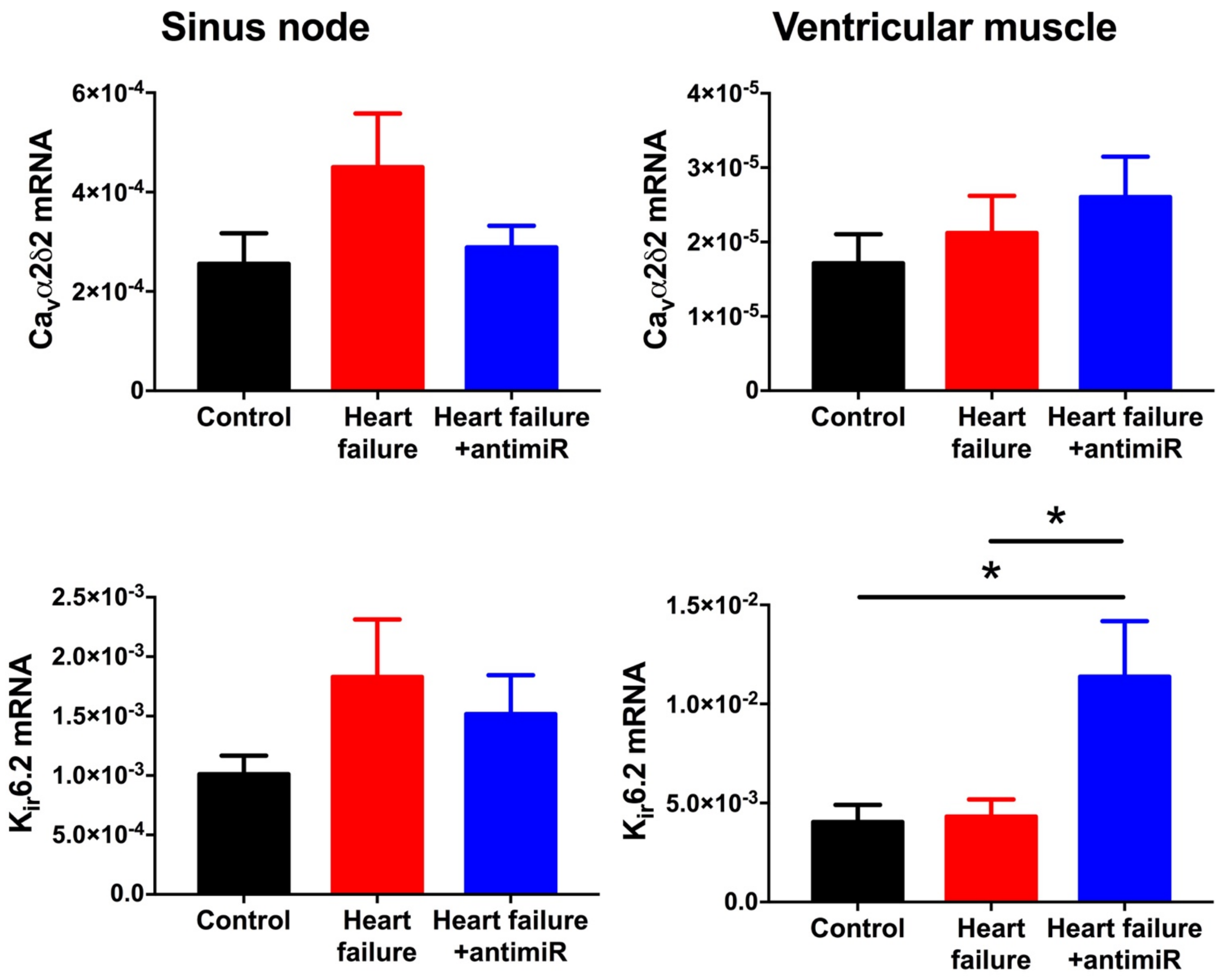

Supplementary Fig. 16. Expression of mRNA of other potential targets of miR-370-3p ($Ca_v\alpha2\delta2$ and $K_{ir}6.2$) in the sinus node and left ventricle of control mice given PBS, heart failure mice given PBS and heart failure mice given antimiR-370-3p (n=9 or 13, 10 or 17 and 5-9). Control and heart failure data same as shown in Fig. 3. *P<0.05; one-way ANOVA followed by Tukey's multiple comparisons test.

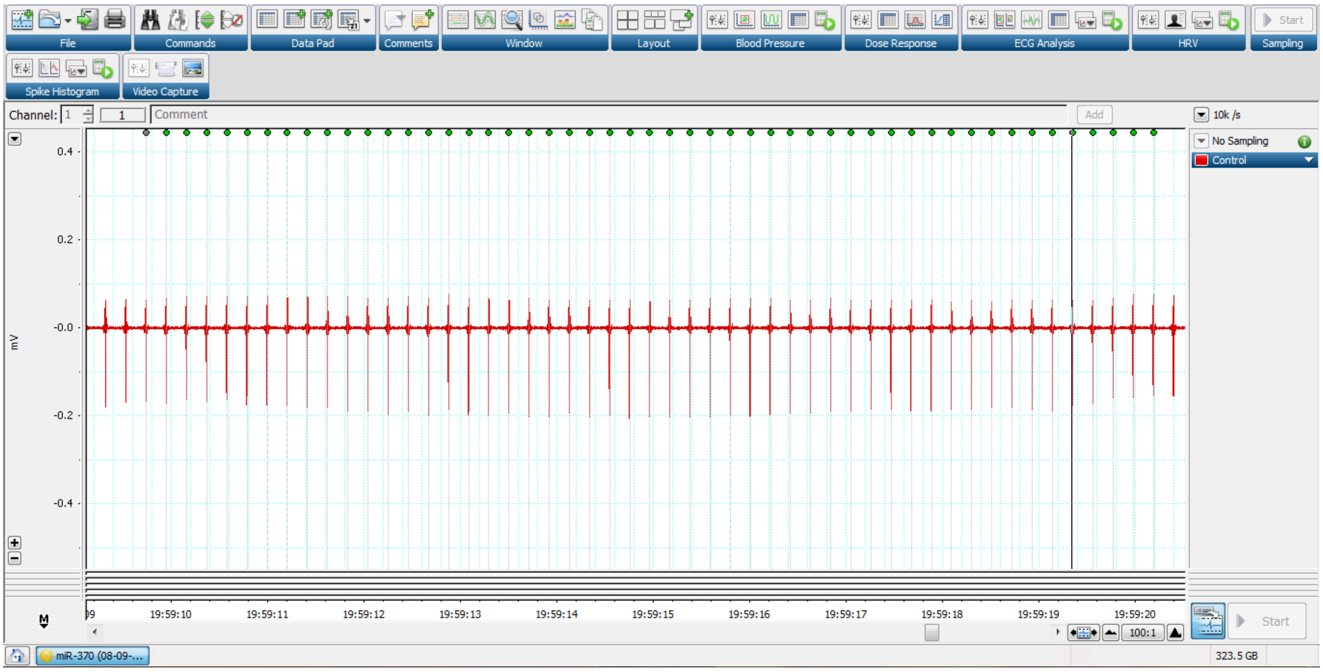

Supplementary Fig. 17. Measurement of beating rate of the isolated sinus node using Powerlab and Chart software (ADInstruments). Screenshot of the Chart software shown. An isolated sinus node preparation was dissected and injected with scrambled microRNA. As described elsewhere, the tissue was maintained in culture medium and the extracellular electrogram recorded. The example screenshot shows an electrogram 8 h after injection (red trace). A beat was identified as a deflection greater than two standard deviations. Green spots show detected beats and they accurately reflect the recorded waveforms.

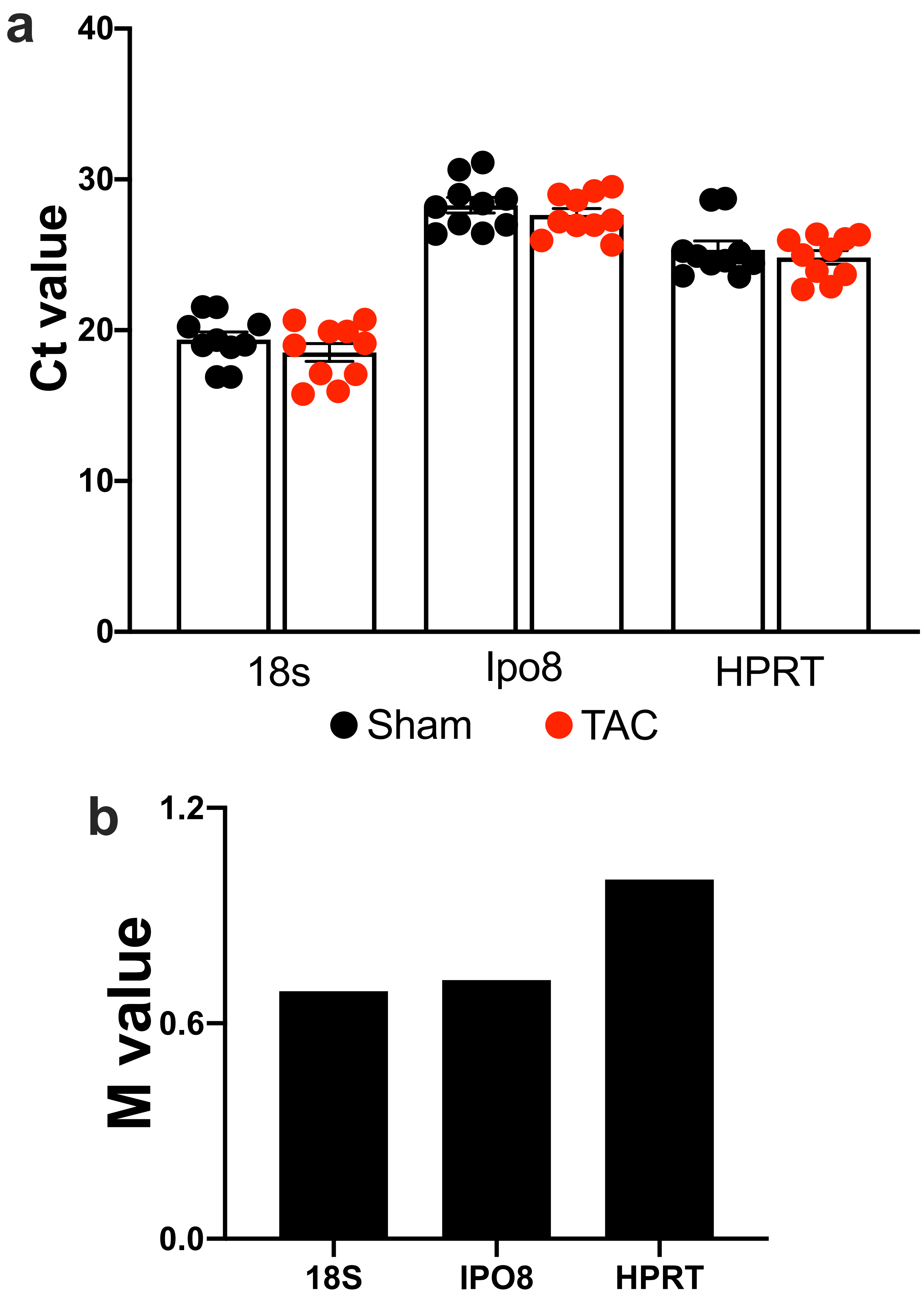

Supplementary Fig. 18. (**a**) Abundance of three reference transcripts in sinus node biopsies from control and heart failure animals (n=10 and 10); Ct values shown. (**b**) M values from GeNorm for the three reference transcripts.
